# Influence of Self-Care on the Quality of Life of Elderly People with Chronic Non-Communicable Diseases: A Systematic Review

**DOI:** 10.3390/healthcare14030308

**Published:** 2026-01-26

**Authors:** Poliana Martins Ferreira, Jonas Paulo Batista Dias, Monica Barbosa, Teresa Martins, Rui Pedro Gomes Pereira, Murilo César do Nascimento, Namie Okino Sawada

**Affiliations:** 1 School of Nursing, Federal University of Alfenas, Alfenas 37130-001, Brazil; murilo.nascimento@unifal-mg.edu.br (M.C.d.N.); namie.sawada@unifal-mg.edu.br (N.O.S.); 2 Ribeirão Preto College of Nursing, University of São Paulo, Ribeirão Preto 14040-902, Brazil; jonas.dias@sou.unifal-mg.edu.br; 3 Porto Nursing School (Escola Superior de Enfermagem do Porto—ESEP), Center for Health Technology and Services Research (CINTESIS@RISE), 4200-072 Porto, Portugal; monicabarbosa.pds@gmail.com (M.B.); teresam@esenf.pt (T.M.); 4 School of Nursing, University of Minho, 4710-057 Braga, Portugal; ruipereira@ese.uminho.pt; 5 Health Sciences Research Unit: Nursing/UMinho, School of Nursing, University of Coimbra, 3046-851 Coimbra, Portugal

**Keywords:** self-care, older adults, chronic non-communicable diseases, quality of life, healthy aging, autonomy

## Abstract

**Background/Objectives:** Self-care is a cornerstone of healthy aging and chronic disease management; however, evidence on the most effective intervention models for improving quality of life in older adults with chronic non-communicable diseases (NCDs) remains fragmented. This review aimed to evaluate the effectiveness of self-care interventions in promoting quality of life and health outcomes in older adults with NCDs. **Methods:** A systematic review was conducted in accordance with PRISMA 2020 guidelines and registered in PROSPERO (CRD420251040613). Randomized and non-randomized clinical trials published between 2019 and 2024 were retrieved from Scopus, Web of Science, and EBSCOhost. Eligible studies included adults aged ≥60 years with NCDs receiving self-care interventions. Data extraction and risk of bias assessment were independently performed using Joanna Briggs Institute tools. **Results:** Twenty-nine studies involving 7241 older adults were included. Self-care interventions comprised nurse-led educational programs, digital health strategies, community- and peer-based approaches, and person-centered care models. Multicomponent and continuous interventions demonstrated consistent improvements in physical and psychological domains of quality of life, self-efficacy, autonomy, symptom management, and treatment adherence. Digital interventions enhanced monitoring and engagement, although their effectiveness varied according to sensory and health literacy limitations. **Conclusions:** Structured, person-centered, and nurse-led self-care interventions are effective in improving quality of life and autonomy among older adults with NCDs. These findings support their integration into primary and community-based care, reinforcing their relevance for clinical practice, care planning, and the development of assistive and educational strategies in aging care.

## 1. Introduction

Quality of life is defined by the World Health Organization (WHO) as “[…] an individual’s perception of their position in life, in the context of the culture and value systems in which they live, and in relation to their goals, expectations, standards, and concerns” [1]. This definition highlights the subjective and multidimensional nature of the concept, encompassing both positive and negative evaluative elements. From a clinical perspective, this multidimensional understanding reinforces the need for interventions that go beyond disease control, incorporating functional, psychological, and social dimensions into care planning for older adults [2].

Interest among social and medical science researchers in quality of life has largely focused on debates regarding its definition and measurement methods [3]. This demographic shift poses significant challenges for health systems, requiring care models that promote autonomy, functional capacity, and self-management among older adults living with chronic conditions [4]. When analyzing how health affects overall well-being and the ability to perform daily activities, the term health-related quality of life is used, a concept more directly associated with diseases or health interventions [5].

Aging is a universal, dynamic, progressive, and gradual process influenced by genetic, biological, social, environmental, psychological, and cultural factors [6]. Interest in issues related to aging has increased in recent decades due to the aging of the global population, an unprecedented demographic phenomenon [7]. Longevity with quality of life has been discussed as an ideal that values not only the extension of life but also the fulfillment of human aspirations [8]. Thus, the promotion of healthy aging emerges not only with a focus on older adults but also as a reference for health practices in general. Although there are typical characteristics of aging, such as gray hair, wrinkled skin, and reduced vigor, associated with senescence [9], the experience of aging varies greatly between individuals. There is no linear correspondence between chronological age and biological age, as aging trajectories are influenced by social, cultural, and historical factors [10,11].

Health occupies a central place in aging due to its direct impact on quality of life, and it is also shaped by stigmas and age-related prejudices. The negative view of old age, historically associated with biological decline, has been widely questioned. Old age has frequently been associated with illness and dependency, erroneously accepted as normal and inevitable characteristics [12]. However, it is essential to distinguish aging from pathology, as they are independent phenomena, and aging may occur as a natural and healthy process [13]. This distinction has important clinical implications, as it supports care approaches focused on functional preservation, empowerment, and active participation of older adults in their own health management [14]. Currently, a significant shift is observed in the perception of old age, with older adults taking on active roles in society, participating in community life, supporting their families financially, and performing various social functions. This perspective breaks the association between aging, dependency, and decline, demonstrating that longevity can be accompanied by autonomy, productivity, and quality of life [15].

Aging can be classified as primary or normal (irreversible, progressive, and universal changes that are not pathological), secondary (changes caused by age-related diseases), and tertiary (terminal decline in advanced old age) [16]. In this context, reflecting on successful aging, also referred to as productive or active aging, represents a critical response to the traditional association between old age, decline, and inactivity [17]. The concept of successful aging emphasizes that aging well involves not only the absence of disease but also the maintenance of high levels of physical and cognitive function, social engagement, and autonomy, and it has been widely investigated in gerontology [18,19].

The adoption of health habits such as physical activity, periodic check-ups, healthy eating, and leisure activities contributes to better quality of life and the prevention of health problems [20]. In clinical and community-based care, these habits are operationalized through structured self-care interventions, often led by health professionals, particularly nurses, aiming to improve treatment adherence, symptom control, and quality of life [21]. These habits promote and sustain self-care, which is essential for clinical stability and well-being in chronic conditions, as the development of self-care knowledge and skills favors autonomy in activities of daily living [22,23].

Self-care encompasses multiple dimensions and, when it includes activities related to religiosity/spirituality and social relationships, it plays an important role in preventing loneliness, grief, stress, and depression [22,24].

The aim of this study was to synthesize evidence on the effectiveness of self-care interventions in improving the quality of life of older adults with chronic non-communicable diseases (NCDs).

The secondary objectives were to: (i) characterize the main self-care intervention models applied to older adults with NCDs; (ii) examine their effects on additional clinical, functional, and psychosocial outcomes; and (iii) analyze how intervention characteristics, delivery modes, and professional support influence intervention effectiveness. Self-care practices are understood as processes undertaken by individuals themselves to maintain, monitor, and manage chronic conditions [22].

## 2. Materials and Methods

The protocol of this review [25] was developed in accordance with the Preferred Reporting Items for Systematic Review and Meta-Analysis Protocols (PRISMA-P) [26] and was registered in the international prospective register of systematic reviews, PROSPERO (CRD420251040613). The reporting of this study was conducted in accordance with the Preferred Reporting Items for Systematic Reviews (PRISMA) [27], when applicable. The completed PRISMA checklist (https://www.prisma-statement.org/prisma-2020-checklist 9 December 2025) is provided as Supplementary Materials (Table S1).

### 2.1. Development of the Guiding Question

The guiding question was developed based on the PICO strategy (Population, Intervention, Comparator, Outcomes), as described in Table 1 [28].

### 2.2. Eligibility Criteria

This review included primary studies with an experimental approach, comprising randomized clinical trials, published between 2019 and 2024, in English or Portuguese, that presented intervention and comparator groups.

Studies were excluded if they did not involve adults aged ≥ 60 years with NCDs, if they were secondary studies (such as reviews or meta-analyses), observational studies, case reports or case series, letters, editorials, or expert opinions.

Older adults (≥60 years) with NCDs were considered eligible participants, regardless of sex, marital status, socioeconomic condition, or geographic context. The age threshold of 60 years was adopted in accordance with the World Health Organization criteria and to ensure comparability between studies conducted in both developed and developing countries [1]. Regarding the type of intervention, this referred to self-care activities used in structured formats, with the aim of improving participants’ quality of life and/or health conditions. Interventions associated with adjunct technologies not directly related to self-care, such as specific pharmacological therapies or surgical interventions, were excluded.

The primary outcomes considered were quality of life and health status, both assessed using validated instruments. There were no restrictions regarding the study setting; participants receiving care in primary, secondary, or tertiary health services were included, as well as those followed in home or community contexts.

The temporal delimitation between 2019 and 2024 was established to ensure the scientific relevance and contemporaneity of the included studies, encompassing research that reflects current self-care practices and their relationship with quality of life among older adults with NCDs. This period also coincides with significant advancements in public policies, the strengthening of primary health care, and the development of educational and technological strategies aimed at self-care in older adults. Additionally, it covers the context of the COVID-19 pandemic, which significantly impacted self-care practices and health outcomes among older adults, making studies from this period particularly relevant for understanding the topic.

The choice of publications in English and Portuguese is justified by the predominance of English as the universal scientific language and Portuguese as the official language of Brazil, broadening international coverage while ensuring representation of studies produced in Portuguese-speaking countries.

### 2.3. Sources of Information and Research Strategy

A comprehensive and reproducible search strategy was developed using controlled vocabularies and free-text terms. Health Sciences Descriptors/Medical Subject Headings (DeCS/MeSH), Medical Subject Headings (MeSH), and CINAHL Subject Headings were consulted to identify relevant terms related to older adults, chronic non-communicable diseases, self-care interventions, and quality of life.

The search strategy combined terms using Boolean operators (AND, OR), with truncation (*) and phrase searching applied where appropriate. An initial search string was developed and subsequently adapted to the syntax and indexing requirements of each database.

Searches were conducted in Scopus (Elsevier), Web of Science Core Collection (Clarivate Analytics), and EBSCOhost (considering all available databases, including CINAHL). The final searches retrieved 85 records from Scopus, 427 records from Web of Science, and 250 records from EBSCOhost. The complete search strategies used for each database are provided in Supplementary Table S2. The complete search strategies used for each database are provided in Supplementary Table S2.

The search strategy used (Supplementary Table S3. Keywords and controlled vocabulary terms used in the search) was adapted to the specific requirements of each information source consulted for this review. Searches were carried out in the following information sources: Scopus (Elsevier), Web of Science—Core Collection (Clarivate Analytics), and EBSCOhost (considering all databases available through the institution, including CINAHL). The literature searches were conducted in November 2024 and subsequently updated in May 2025 to ensure the identification of all eligible studies published within the predefined 2019–2024 time frame.

### 2.4. Study Records

#### 2.4.1. Data Management

The results obtained from the databases were exported to EndNote (EndNote Web, Clarivate, Philadelphia—https://www.myendnoteweb.com) [29], and duplicate articles were removed. Subsequently, the articles were uploaded to the Rayyan software for the study selection process [30].

#### 2.4.2. Selection Process

The study selection was carried out through the assessment of titles, abstracts, and keywords according to the eligibility criteria. Relevant articles were read in full, and those that did not meet the predetermined criteria for this review were excluded. The study selection process was conducted independently by two reviewers (R1 and R2). In cases of inconsistencies, these were discussed and resolved, and when necessary, a third reviewer was involved to reach consensus. The reviewers were not blinded to the journal of publication, authors, or institutions at any stage of the selection process. The selected materials were saved in full in a folder for subsequent analyses.

#### 2.4.3. Data Extraction

Data extraction was performed using a standardized extraction form developed for this purpose. The instrument included information from each study, such as authorship, year of publication, study design, objective, population, self-care activity under analysis, other interventions, main results, and conclusions.

The extraction process was conducted independently by two reviewers (R1 and R2). Subsequently, the results were compared and discussed jointly with a third reviewer (R3), ensuring consensus and the inclusion of only the information considered relevant in the final form. Prior to the final extraction, a pilot test of the data extraction form was conducted using a subset of the included studies to assess clarity, consistency, and applicability of the instrument. Based on this pilot phase, minor refinements were made to standardize terminology and data categorization. One of the authors (R1) subsequently reviewed the final version of the data extraction table to ensure uniform data presentation and to identify any missing or incomplete information.

All information was systematized in comparative tables constructed from the included studies, enabling critical analysis and synthesis of the available evidence, in accordance with the methodological recommendations of the Joanna Briggs Institute (JBI) for systematic reviews of experimental studies. The complete data extraction table, including the variables author–year, study objective, method, population (n; chronic diseases), self-care activity, comparator, quality of life outcomes, key findings, and reported limitations, is provided as Supplementary Table S4.

#### 2.4.4. Data Synthesis

Data synthesis was conducted in a descriptive and narrative manner, based on the information extracted from the studies included in this review. This systematization allowed for the comparison of results, identification of effectiveness patterns, as well as the recognition of knowledge gaps and implications for clinical practice and research in older adult health.

Due to the heterogeneity of the included studies, particularly regarding the proposed self-care interventions, measurement instruments, and evaluated outcomes, it was not possible to perform a meta-analysis. Therefore, the findings were presented in structured comparative tables and narrative synthesis, in accordance with the methodological recommendations of the Joanna Briggs Institute (JBI) for systematic reviews of experimental studies.

#### 2.4.5. Critical Appraisal of the Studies

Two reviewers critically appraised the eligible studies regarding methodological quality. We used the Joanna Briggs Institute Critical Appraisal Checklists for randomized clinical trials [31]. The purpose of this appraisal was to assess the methodological quality of each study and determine the extent to which it addressed the possibility of bias in its design, conduct, and analysis [31]. We assigned ratings of yes (Y), no (N), unclear (U), and not applicable (N/A) for each criterion in these checklists. Discrepancies were resolved through joint discussion with the authors to reach consensus.

This review included only randomized clinical trials, that were appraised using the Joanna Briggs Institute (JBI) critical appraisal tools appropriate for this methodology.

## 3. Results

In total, the sample consisted of 29 articles. The table with the data extracted from each study is available as Supplementary Materials.

**Figure 1:**
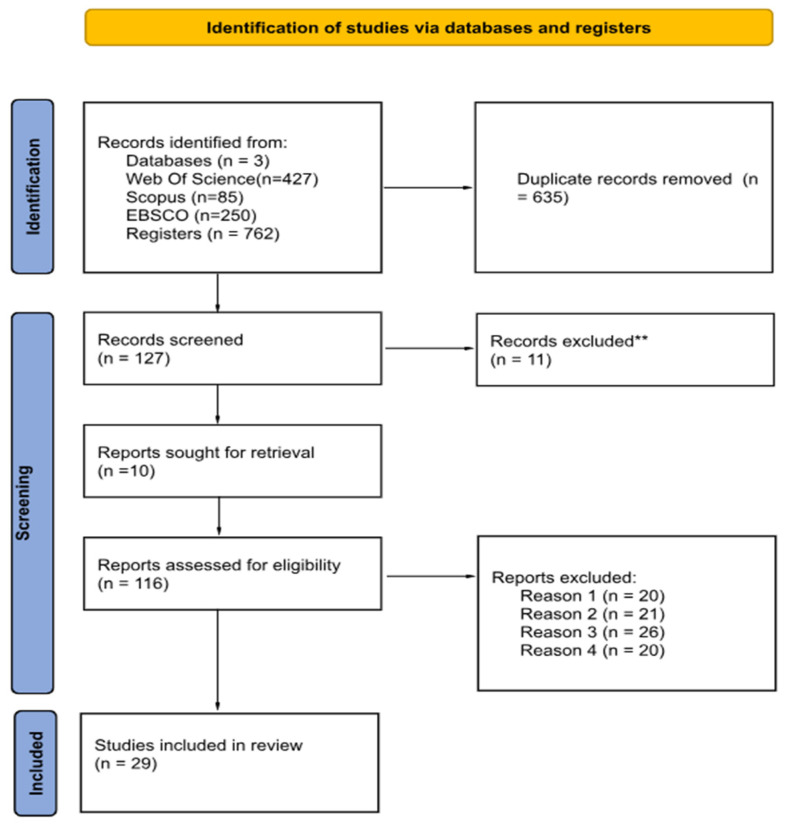
PRISMA 2020 flow diagram [27]. Notes: Reason 1 = Does not involve older adults; Reason 2 = Publication type does not meet the defined eligibility criteria; Reason 3 = Study design does not meet the defined eligibility criteria; Reason 4 = Does not address self-care practices. ** indicate how many records were excluded.

The study selection process is presented in Figure 1. The included studies were published between 2018 and 2024, and among them, 23 (79.3 percent) were published from 2020 onward, reflecting the significant growth of research on self-care, quality of life, and healthy aging in the last decade.

A total of 7241 older adults participated in the 29 clinical trials included in this review, all involved in self-care interventions for individuals with NCDs. The mean age of participants ranged from 60 to 78 years, with a predominance of women (approximately 69 percent) and older adults who were retired or had low educational levels, residing in the community.

Regarding geographic distribution, 41.3 percent of the studies (*n* = 12) were conducted in Asian countries (China, Japan, Iran, Singapore, and Hong Kong) [32,33,34,35,36,37,38,39,40,41,42,43]. Twenty-eight percent of the studies (*n* = 8) were conducted in North America [44,45,46,47,48,49], 24 percent (*n* = 7) in Europe [50,51,52,53,54], and 7 percent (*n* = 2) in Oceania [55,56].

With respect to the chronic conditions addressed, ten studies (34.5 percent) included older adults with type 2 diabetes mellitus [32,33,34,42,43,57]. Six studies (20.7 percent) investigated cardiovascular diseases, including hypertension and heart failure [39,40,41,54]. Three studies (10.3 percent) addressed Chronic Obstructive Pulmonary Disease (COPD) [37,52,53], and another three (10.3 percent) involved older adults with multimorbidity [46,47,50]. Two studies (6.9 percent) focused on mental health conditions, particularly persistent depression [51,58], and two studies (6.9 percent) involved older adults with cancer or in post-oncological treatment [36,55]. The remaining three studies (10.3 percent) included populations with low vision [31], post-stroke individuals [56], and healthy aging with risk of NCDs [59].

Regarding the type of intervention, seventeen studies (58.6 percent) implemented nurse-led educational self-care programs, often based on models such as Social Cognitive Theory and Person-Centered Care [32,46,47,51]. Six studies (20.7 percent) used digital technologies, including mobile applications and telemonitoring [38,39,40,48,49,56]. Four studies (13.8 percent) explored community support or peer-led interventions [45,50,51,59]. Two studies (6.9 percent) were grounded in the Roy Adaptation Model [41,54].

The main self-care practices identified were clinical self-management (medication adherence, blood glucose control, and blood pressure monitoring), healthy eating, regular physical activity, symptom monitoring, stress and emotion management, use of digital technologies, and strengthening of family and community support networks.

The duration of the interventions ranged from 8 weeks to 12 months, with an average follow-up of 6 months, and most combined in-person sessions with remote follow-up, either by telephone or digital means. The most effective interventions involved continuous nursing support and participatory education, with an emphasis on autonomy, self-efficacy, and the shared responsibility of older adults in managing their own health, Table 2.

### 3.1. Integrated Overview of the Effectiveness of Self-Care Interventions Among Older Adults

#### 3.1.1. Physical Activity

Trials using structured exercise, muscle-resistance training for older adults with cancer [36] and Tai Chi for individuals with multiple chronic conditions [44] showed significant improvements in strength, mobility, cancer-related fatigue, and blood pressure control. However, the impact on quality of life (QoL) was inconsistent: resistance training improved vitality and mental state [36], whereas the Tai Chi program did not outperform health education at 6 and 12 months [44]. These findings suggest that structured exercise improves clinical outcomes, though perceived QoL varies according to intensity and duration. Structured exercise interventions primarily improved physical and functional outcomes; however, their effects on quality of life were inconsistent. These findings suggest that physical gains alone may be insufficient to influence broader perceptions of well-being, highlighting the need to integrate exercise with educational and psychosocial support to enhance quality-of-life outcomes in older adults.

#### 3.1.2. Health Education and Self-Management

Self-care–focused interventions for diabetes [32,33,43]), low vision [35], chronic conditions in primary care [42], hypertension based on the Roy Adaptation Model [53], and multimorbidity [51] demonstrated consistent improvements in self-management, self-efficacy, medication adherence, and health knowledge. Significant gains in QoL—especially emotional well-being, energy, and social functioning, were reported in multiple trials [32,33,35,42,53,57], including those addressing persistent depressive disorder [57]. Programs explicitly grounded in theoretical models, such as the Roy Adaptation Model, had strong effects on blood pressure, adherence, and multiple SF-36 domains [53]. However, trials with adults with advanced multimorbidity exhibited more modest QoL improvements, likely due to greater clinical complexity [56]. Health education and self-management programs showed the most consistent improvements in quality of life, particularly in emotional well-being and autonomy. Interventions grounded in theoretical models strengthened self-efficacy and adherence, supporting their relevance for clinical practice, especially when tailored to individuals with complex or multiple chronic conditions.

#### 3.1.3. Digital Technologies and mHealth

Digital interventions: including mobile apps with nurse support [38], nurse-led telemonitoring for heart failure [39], home-based telemonitoring [40], web-based self-management platforms [51], and remote coaching for stroke survivors [60], consistently yielded improvements in QoL, particularly in the mental health dimension. These studies also demonstrated reductions in decompensation episodes and rehospitalizations, especially among patients with heart failure [38,39,40]. Interventions involving structured professional support, such as nursing follow-up, produced stronger and more sustained effects [38,39,55]. In contrast, interventions based solely on text messaging, with minimal professional engagement, yielded more modest outcomes [54,60]. Overall, digital interventions were more effective when supported by continuous clinical guidance [61,62]. Digital interventions improved quality of life mainly when combined with continuous professional support. Nurse-led telemonitoring and guided digital tools enhanced engagement and mental health outcomes, whereas stand-alone technological approaches showed limited effects, underscoring the importance of human support in technology-mediated care for older adults.

#### 3.1.4. Collaborative, Community-Based, and Transitional Models

Collaborative management interventions improved self-management, communication, satisfaction with care, and depressive symptoms, as shown in trials with couple-based diabetes management [34], coronary heart disease using a dual-track interactive nursing model [41], and transitional nurse-led care for older adults with multimorbidity and depressive symptoms [46]. Collaborative telemonitoring programs in heart failure also demonstrated reductions in rehospitalizations and improvements in QoL [39,40]. However, highly complex personalized care models, such as LoChro-Care, did not produce improvements in QoL or functional health, suggesting that intensity, focus, and personalization are decisive factors [54]. Overall, collaborative interventions consistently improved emotional health, continuity of care, and patient engagement, though objective clinical effects varied [34,39,40,46,49]. Collaborative and transitional care models improved emotional health, patient engagement, and continuity of care. Interventions involving families and interdisciplinary teams were more effective than highly complex personalized models, indicating that feasibility and focused coordination are critical for quality-of-life benefits.

#### 3.1.5. Psychosocial and Emotional Interventions

Psychosocial trials focusing on emotional support, spirituality, social participation, and reduction in depressive symptoms also showed strong effectiveness. Spirituality and social activity–based interventions generated improvements in emotional well-being [50], while proactive self-care programs led by nurses improved anxiety, depression, and life satisfaction [58]. Peer-led coaching for chronic pain demonstrated significant increases in pain self-efficacy, mood, and social engagement [59]. Despite these psychological benefits, effects on physical health were generally modest, suggesting that such interventions act predominantly in the emotional and psychosocial sphere of QoL [63,64]. Psychosocial interventions demonstrated strong positive effects on emotional well-being, self-efficacy, and social participation, despite modest physical health changes. These findings reinforce the importance of addressing emotional and social dimensions of care when aiming to improve quality of life in older adults.

#### 3.1.6. Innovative Self-Care Strategies

Innovative models, including campaigns based on advertising principles [48], technologically supported coaching programs [47], and multimodal adherence strategies, showed modest improvements in QoL and moderate reductions in specific clinical events. The advertising-based cardiovascular intervention involving more than 4000 older adults significantly reduced cardiovascular events but did not improve QoL [48]. Programs combining coaching with home devices, such as VADAC and Stroke Coach, produced gains in depression, self-efficacy, social functioning, and general health [47,55]). Innovative self-care approaches yielded selective benefits, often improving specific clinical or behavioral outcomes without consistent gains in quality of life [61,62]. Strategies combining technology with personalized support showed more promising psychosocial effects, suggesting that innovation is most effective when integrated into supportive care frameworks.

In summary, these approaches tend to enhance specific clinical or behavioral indicators, while their effect on subjective QoL is more limited (Table 3).

### 3.2. Critical Evaluation of Studies

**Figure 2:**
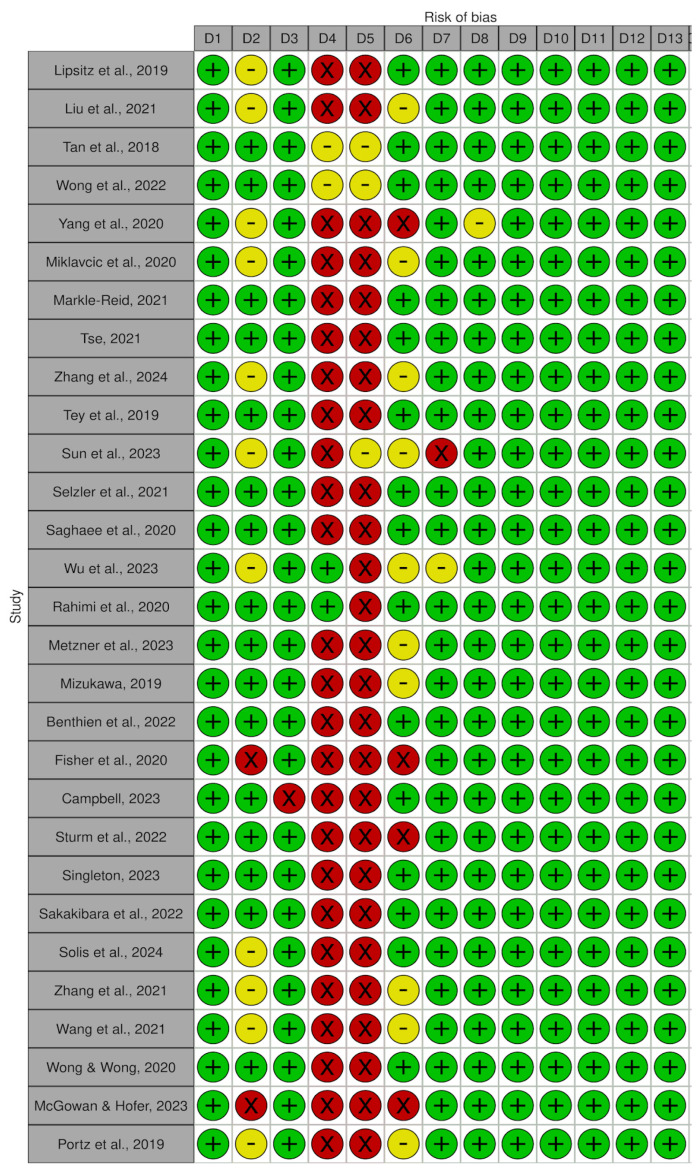
Below presents the traffic light chart that summarizes the risk of bias assessment of the studies analyzed. Legend: 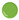 (+) low risk of bias; 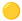 (-) some concerns; 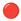 (×) high risk of bias. Risk of bias assessment of the included studies according to JBI criteria (traffic light chart) [32,33,34,35,36,37,38,39,40,41,42,43,44,45,46,47,48,49,50,51,52,53,54,55,56,57,58,59,60].

Although most of the included studies demonstrated high methodological quality, a portion of them presented a moderate risk of bias, which should be considered when interpreting the findings. These methodological aspects may influence the robustness of the conclusions and should be taken into account when evaluating the strength of the available evidence (Figure 2).

## 4. Discussion

An analysis of 29 randomized clinical trials on self-care interventions in older adults with chronic conditions reveals a complex and multifaceted picture of the effectiveness of these approaches. The analysis of the clinical trials included in this review demonstrates that self-care interventions applied to older adults with NCDs are predominantly implemented in primary care and community settings, reinforcing the central role of these networks in maintaining functionality and autonomy in old age. The effectiveness of the interventions studied is more evident in improving clinical aspects than in the perception of quality of life. This difference indicates that self-care interventions tend to be more effective in altering objective clinical parameters than in improving subjective perceptions of well-being. Thus, future programs should go beyond clinical outcomes and incorporate components that promote the holistic well-being of older adults.

The most effective interventions proved to be multicomponent, synergistically combining education, exercise, emotional support, continuous monitoring, and professional coordination. Complex models, such as the “two-way” interactive nursing model [43], which horizontally integrate community, primary care team, family, and patient, and vertically hospital specialists and specialized nurses, have demonstrated significant reductions in anxiety and depression, as well as broad improvements in quality of life. Similarly, enhanced rehabilitation programs [52], which combine physical training, interactive education, SMART goals, and self-management, have produced robust clinical benefits while substantially reducing the use of health services, indicating systemic efficiency gains.

These findings converge with evidence that structured interventions—mostly conducted by nurses and grounded in models such as Social Cognitive Theory and Person-Centered Care—are particularly effective. The combination of ongoing professional support with participatory educational strategies strengthens self-efficacy and sustains adherence to self-care among older adults with chronic conditions, enhancing both clinical outcomes and the autonomy and well-being of participants.

The diversity of chronic conditions addressed—including diabetes, cardiovascular disease, COPD, multimorbidity, and depression—demonstrates that structured self-care practices produce predominantly clinical impacts, although they also promote psychosocial gains. In older adults with type 2 diabetes, for example, self-management and health education interventions were associated with better glycemic control, reduced depressive symptoms, and improved quality of life [65,66]. Among cardiovascular patients, a multicenter study with 5964 participants from 15 countries demonstrated that strengthening self-care improves the early detection of signs of decompensation and therapeutic adherence [67]. Similarly, in interventions targeting older adults with COPD or multimorbidity, self-care programs favored symptom management, energy conservation, and maintenance of social participation, results consistent with the literature on aging and respiratory diseases [68]. Thus, it is observed that, despite benefiting multiple dimensions of the elderly person’s life, self-care interventions achieve their most consistent effects in the clinical sphere, especially when they integrate educational, behavioral, and continuous monitoring components.

Interventions combining technology with structured human support yielded the best results. When technology acted as a facilitator of interaction—whether through personalized Artificial Intelligence coupled with in-person sessions, telephone support operated by trained professionals, or peer coaching complemented by home devices—the effects on emotional health, self-efficacy, and well-being were substantial. In contrast, technological solutions offered without consistent human support showed low adherence and limited impact, especially among older adults, indicating that technology, in isolation, does not sustain engagement or generate significant improvements in quality of life.

Another relevant finding of this review concerns the increasing use of digital technologies, which accounted for 20.7 percent of the evaluated interventions. Tools such as mobile applications, telemonitoring systems, and interactive platforms supported daily monitoring, individualized education, and remote assistance, enhancing older adults’ engagement in managing their own condition, especially among those with reduced mobility or multiple comorbidities. International evidence supports this potential, showing that digital interventions can improve treatment adherence, reduce depressive symptoms, and optimize clinical indicators in populations with NCDs [69,70]. However, some included studies indicated that low educational levels and sensory limitations, which are common among older adults, may restrict the effectiveness of these technologies, suggesting the need for multimodal approaches and complementary in-person support.

In addition to technological interventions, strategies based on community support, group interactions, and peer-led programs showed positive effects on autonomy, self-efficacy, and social engagement among older adults. Recent evidence highlights that peer support plays a decisive role in the self-care of older adults with chronic diseases, as sharing experiences strengthens self-efficacy and helps overcome emotional barriers related to disease management [71]. Likewise, community-based interventions have demonstrated positive effects on autonomy and social engagement, elements that are essential for preserving quality of life during the aging process [72]. In the studies analyzed, programs that incorporated emotional, relational, and social dimensions of self-care achieved broader improvements in psychological domains and perceived well-being, reinforcing that self-care is a multidimensional phenomenon that goes beyond individual actions and is built through support networks.

The involvement of family members and caregivers proved crucial to the success of the interventions. Studies indicate that family management models, with home visits, shared education, and active family participation, improve glycemic control, treatment adherence, and reduce anxiety and depression. Furthermore, family members act as continuous educational reinforcement, increasing knowledge about the disease. Programs that systematically integrated the family showed fewer complications and lower dropout rates, even when the clinical effects were not statistically significant [43,54].

From a clinical and care-oriented perspective, this review demonstrates that effective self-care interventions for older adults with chronic non-communicable diseases extend beyond disease control, requiring the integration of functional, psychological, and social dimensions of care. Interventions combining structured health education, continuous professional support, and active patient engagement were consistently associated with improvements in quality of life, particularly in emotional well-being, autonomy, and self-efficacy [1,2,3,4].

These findings have direct implications for primary care and community-based practice, where nurse-led and interprofessional models play a central role in supporting self-care, monitoring symptoms, and coordinating care. The greater effectiveness of interventions with sustained professional follow-up highlights that self-care should be conceptualized as a supported and dynamic process rather than an isolated individual responsibility [1,5,6,7].

From an assistive care perspective, tailoring interventions to functional capacity, health literacy, and psychosocial needs is essential. Digital and mHealth strategies showed benefits when embedded within supportive care models, whereas stand-alone technological interventions yielded more limited effects [8,9,10,11]. Overall, the findings support the integration of self-care interventions into routine, person-centered, and longitudinal care frameworks, aligned with contemporary models of chronic care and healthy aging that prioritize continuity of care and quality of life [12,13,14,15].

### 4.1. Perspectives for Clinical and Care Practice

From a clinical and care-oriented perspective, the findings of this review reinforce that effective self-care interventions for older adults with chronic non-communicable diseases must extend beyond disease control and incorporate functional, psychological, and social dimensions of care. This multidimensional understanding of quality of life is consistent with the conceptual framework proposed by the World Health Organization, which emphasizes physical, psychological, social, and environmental domains as core components of well-being [1]. Evidence from international systematic reviews demonstrates that self-management interventions supported by continuous professional follow-up are associated with greater improvements in self-efficacy, autonomy, and health-related quality of life, particularly when grounded in structured educational approaches and theoretical models of chronic care [2,24]. Nurse-led and interprofessional models, in particular, have shown effectiveness in promoting continuity of care, symptom monitoring, and patient engagement in primary care and community settings [24,30,56]. From an assistive care perspective, digital and mHealth interventions appear to yield meaningful benefits when embedded within supportive care frameworks; however, stand-alone technological solutions tend to show limited effectiveness, especially among older adults with lower health literacy or functional limitations [6]. These findings support the integration of self-care interventions into routine, person-centered, and longitudinal care models, aligned with contemporary frameworks of healthy aging and chronic disease management [1,4,25].

This review has some limitations that should be acknowledged. First, most of the included studies did not report information on the costs or cost-effectiveness of the self-care interventions, which limits the assessment of their feasibility and scalability in different healthcare settings. Second, quality of life and other outcomes were predominantly measured using self-reported instruments, which may be subject to recall bias and social desirability bias. Additionally, there was considerable heterogeneity among the included studies regarding intervention components, duration, intensity, follow-up periods, and outcome measures, which limits direct comparisons and precludes more robust quantitative synthesis. Variability in sample sizes and clinical complexity of the populations studied, particularly among individuals with advanced multimorbidity, may also have influenced the magnitude of the observed effects. Finally, the predominance of studies conducted in high-income countries restricts the generalizability of the findings to low- and middle-income settings. Moreover, the inclusion of studies published only in English and Portuguese may have resulted in the exclusion of relevant evidence published in other languages. In addition, despite the high prevalence of dementia and other cognitive impairments among older adults, these conditions were underrepresented in the included studies, highlighting an important gap for future research. These limitations should be considered when interpreting the results and highlight the need for future studies with standardized outcomes, longer follow-up, economic evaluations, and broader geographic representation.

Despite the positive findings, the methodological heterogeneity of the studies, variability in the qualifications of the professionals involved, and lack of standardization of core intervention components limit direct comparisons and the identification of the most influential elements for improving quality of life. Additionally, although some studies demonstrated additional clinical benefits, such as reductions in blood pressure, improvements in glycemic control, or decreases in exacerbations, these results were less consistent and strongly dependent on older adults’ adherence, reinforcing that self-care mediates the clinical impact of interventions. Such limitations have been widely discussed in international systematic reviews, which emphasize the need for more robust protocols, standardized measurement, and longitudinal follow-up to strengthen the evidence base in this field [68,69]. Even so, the body of evidence shows that self-care interventions, when well structured, continuous, and person-centered, represent an essential strategy for promoting quality of life and autonomy in older adults with NCDs.

### 4.2. Strengths and Limitations

O The review protocol was developed according to PRISMA-P, and reporting followed PRISMA 2020, ensuring methodological rigor and transparency. The use of Joanna Briggs Institute (JBI) critical appraisal checklists and the independent participation of reviewers in study selection, data extraction, and appraisal strengthened the reliability of the findings. The comprehensive search strategy, covering multiple international databases and publications in English and Portuguese, enhanced the scope and representativeness of the evidence.

This study may present some limitations. The heterogeneity of the included studies, particularly regarding intervention types, measurement instruments, and follow-up duration, limited direct comparability across trials. Although most studies demonstrated high methodological quality, some presented a moderate risk of bias, which should be considered when interpreting results. Variability in the training of professionals and the lack of standardization of intervention components may have influenced outcome consistency. Additionally, despite the extensive search strategy, some relevant studies may not have been identified, and the possibility of publication bias cannot be fully excluded.

## 5. Conclusions

This systematic review demonstrated that structured and continuous self-care practices, particularly those mediated by nurses and grounded in self-management and person-centered care models, support improvements in the quality of life of older adults with non-communicable chronic diseases. Interventions that combine health education, family or community support, digital technologies, and regular follow-up showed greater adherence and strengthened autonomy in the management of one’s own health. However, the heterogeneity of study designs, measurement instruments, and intervention duration limits the comparability of results and the precise identification of the most effective components. Thus, there is a need for robust clinical trials with longer follow-up periods and the use of standardized measures to assess quality of life and self-care competencies.

## Figures and Tables

**Table 1:** Development of the guiding question based on the PICO strategy (Population, Intervention, Comparator, Outcomes).

PICO	Components
Guiding question	How effective are the different self-care intervention in promoting quality of life and improving health outcomes among older adults with chronic non-communicable diseases?
P—Population	Adults aged ≥60 years living with NCDs (e.g., cardiovascular disease, diabetes, COPD, cancer).
I—Intervention	Structured self-care intervention
C—Comparator	Usual care, standard care, or alternative self-care interventions
O—Outcome	Quality of life and health outcomes

**Table 2:** Summary of self-care intervention studies and their impacts on quality of life and health outcomes in older adults.

Author–Year	Main Condition(s)	Type of Self-Care Intervention	QoL Impact	Health Impact
Lipsitz et al., 2019 [44]	Multimorbidity	Tai Chi + health education	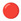	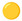
Liu et al., 2021 [34]	Type 2 diabetes	Couple-based collaborative self-care	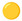	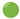
Tan et al., 2020 [32]	Type 2 diabetes	Self-efficacy–based education program	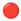	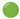
Wong et al., 2022 [38]	Hypertension, diabetes, chronic pain	mHealth with nurse support	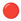	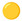
Yang et al., 2020 [41]	Coronary heart disease	Two-way interactive nursing model	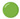	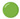
Miklavcic et al., 2020 [56]	Multimorbidity	Community-based self-management	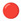	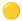
Markle-Reid et al., 2021 [46]	Multimorbidity + depression	Nurse-led hospital-to-home transition care	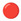	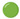
Tse et al., 2021 [59]	Chronic musculoskeletal pain	Peer-led pain self-management	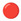	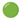
Zhang et al., 2024 [42]	Hypertension, diabetes	Person-centered health education	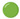	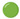
Tey et al., 2019 [35]	Low vision	Self-management and coping strategies	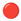	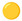
Sun et al., 2023 [36]	Cancer	Supervised resistance exercise	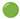	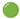
Selzler et al., 2021 [52]	COPD	Pulmonary rehabilitation + self-management	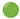	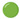
Saghaee et al., 2020 [33]	Type 2 diabetes	Structured diabetes self-management education	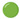	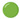
Rahimi et al., 2020 [40]	Heart failure	Home monitoring without sustained support	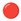	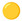
Metzner et al., 2023 [49]	Advanced multimorbidity	Personalized collaborative care (LoChro-Care)	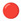	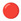
Mizukawa et al., 2019 [39]	Heart failure	Nurse-led telemonitoring	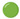	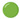
Wu et al., 2023 [37]	Multiple chronic diseases	Community health education + monitoring	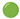	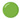
Benthien et al., 2022 [51]	High hospitalization risk	Telephone-based nurse self-care support	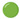	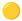
Fisher et al., 2020 [45]	Multimorbidity	Community self-management program	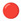	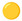
Campbell et al., 2023 [48]	High cardiovascular risk	Self-care support using advertising principles	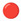	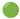
Sturm et al., 2022 [50]	Multiple chronic diseases	Spirituality, self-care, and social support	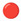	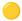
Singleton et al., 2023 [54]	Breast cancer survivors	Lifestyle-focused SMS intervention	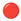	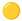
Sakakibara et al., 2022 [55]	Stroke	Telecoaching (Stroke Coach)	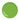	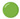
Solis et al., 2024 [57]	Persistent depression	Self-management with caregiver involvement	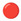	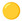
Zhang et al., 2021 [53]	Hypertension	Roy Adaptation Model nursing care	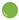	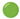
Wang et al., 2021 [43]	Type 2 diabetes	Family- and organization-based management	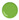	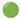
Wong & Wong, 2020 [58]	Multiple chronic diseases	Proactive nurse-led self-care	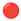	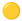
McGowan & Hofer, 2022 [47]	Mixed chronic conditions	Peer coaching ± electronic devices	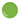	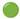
Portz et al., 2019 [60]	Type 2 diabetes and other chronic conditions	Type 2 diabetes and other chronic conditions	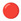	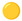

Legend: 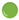 significant improvement; 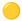 partial/moderate; 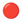 no significant improvement.

**Table 3:** Overview of self-care intervention models and their reported effects on quality of life and health outcomes among older adults with chronic non-communicable diseases.

Self-Care Intervention Model	Main Components	Overall Impact on QoL	Overall Impact on Health
Educational self-care programs (nurse-led, structured)	Health education, skills training, self-management support	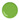 Significant improvement, especially in physical and emotional domains	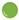 Improved clinical indicators and treatment adherence
Digital and mHealth interventions	Apps, telemonitoring, web-based platforms, SMS	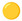 – 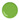 Moderate to significant improvement, stronger with professional support	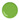 Reduced hospitalizations and improved disease control
Physical activity–based interventions	Tai Chi, resistance exercise, pulmonary rehabilitation	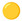 Variable impact depending on intensity and duration	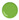 Improved strength, mobility, and symptom control
Collaborative and transitional care models	Interprofessional care, family involvement, nurse coordination	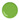 Consistent improvement, particularly in emotional well-being	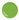 Improved continuity of care and reduced rehospitalization
Psychosocial and peer-based interventions	Spirituality, peer coaching, social participation	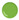 Strong improvement in emotional and psychosocial QoL domains	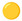 Limited direct clinical impact
Innovative and technology-supported strategies	AI education, social marketing, automated messaging	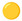 Modest or domain-specific improvements	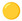 Selective clinical benefits

Legend: 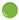 significant positive impact; 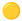 moderate or partial impact.

## Data Availability

No new data were created or analyzed in this study.

## Supplementary Materials

The following supporting information can be downloaded at: https://www.mdpi.com/article/10.3390/healthcare14030308/s1, Table S1: Completed PRISMA 2020 checklist; Table S2: Complete search strategies used for each database (Scopus, Web of Science, and EBSCOhost); Table S3: Keywords and controlled vocabulary terms used in the search strategy; Table S4: Data extraction table of the included studies.
