# Supplementary material for: Influence of Self-Care on the Quality of Life of Elderly People with Chronic Non-Communicable Diseases: A Systematic Review
Source: Healthcare (Basel). 2026 Jan 26;14(3):308. doi: 10.3390/healthcare14030308 (PMC12896789; doi:10.3390/healthcare14030308)
Supplement: Supplementary file 1 [file healthcare-14-00308-s001.zip › healthcare-4065740-supplementary.pdf]

Table S4: Data extraction table of the included studies.

Author-year	Study objective	Method	Population (n; chronic diseases)	Self-care activity	Comparator	Results in Quality of Life	Key findings	Limitations
Lipsitz et al., 2019	To test the hypothesis that Tai Chi improves physical function in elderly people living in low-income housing.	RCT by clusters	n=180; hypertension, diabetes, osteoarthritis , heart disease, COPD	Group Tai Chi practice	Health education	SF-12: no improvement in functional health ($p \geq 0.05$)	The practice did not result in improved quality of life or functional capacity.	Contamination between groups; variable adherence; no blinding.
Liu et al., 2021	To systematically develop and validate a collaborative, couple-based management model that integrates healthcare professionals and supportive	RCT pilot	n=18 couples; type 2 diabetes	Collaborative management in pairs	Individual management	SF-36: significant improvement in the physical component ($p = 0.04$); no improvement in the mental component ($p > 0.05$)	It improved physical aspects of quality of life.	Due to the small sample size and short duration, the study lacked statistical power.

	family members.							
Tan et al., 2020	Evaluate the program to improve self-efficacy in diabetes management (DSEEP).	RCT	n=113; type 2 diabetes	DSEEP Program (education and reinforcement)	Usual care	ADDQoL : there was no significant difference in quality of life ($p \geq 0.05$)	The program increased self-efficacy , which successfully improved self-care activities and reduced HbA1c.	Self-report ; no blinding; high dropout rate.
Wong et al., 2022	To evaluate the effects of an interactive mobile health (mHealth) program on the quality of life (QoL) of older adults living in the community.	RCT	n=221; hypertension, diabetes, and chronic pain	Nursing app and monitoring	He did not receive the application or health-social services.	SF-12: no difference ($p \geq 0.05$)	No significant improvements were found in quality of life (QoL).	Limited use of technology; nurse assistance only during office hours.
Yang et al., 2020	To evaluate the effect of the interactive "two-way" nursing model on anxiety,	RCT	n=103; coronary heart disease	Education in self-care, emotional support, guided exercises,	Usual care	SF-36: significant improvement ($p<0.01$)	Comprehensive improvement in quality of life and symptoms of depression and anxiety.	The intervention is short in duration and follow-

	depression, and quality of life in patients with coronary artery disease.			symptom diary, and communication with nurses.				up is limited.
Miklavcic et al., 2020	To evaluate the effectiveness of a community-based self-care program in improving clinical and psychosocial outcomes in older adults with type 2 diabetes and multiple chronic conditions.	Pragmatic RCT	n=132; DM2; hypertension, dyslipidemia, chronic musculoskeletal pain, cardiovascular disease, osteoarthritis .	Guidelines for symptom management, diet, exercise, medication use, goal setting, and social support.	Usual care	SF-12: no difference (p≥0.05)	The program increased engagement and self-confidence in self-care, but did not result in improved quality of life.	self-reported data and the unavailability of clinical outcome data.
Markle-Reid et al., 2021	To evaluate the effectiveness of a nurse-led hospital-to-home transition intervention in	Pragmatic RCT	n=127; cardiovascular disease, type 2 diabetes mellitus, chronic lung	Home visits, ongoing telephone support, medication reconciliation, self-care	Usual care	VR-12: There was no significant improvement in quality of	It reduced unplanned visits to the emergency department.	Heterogeneous population; variable adherence; follow-up time may

	improving health outcomes in older adults with multiple chronic diseases and depressive symptoms.		disease, osteoarthritis , chronic musculoskeletal pain, and depressive symptoms	education, personalized care plan, and coordination with community services.		life in the physical and mental scores (p> 0.05).		have been short.
Tse et al., 2021	To evaluate the effectiveness of a peer-led pain management program in reducing chronic pain and strengthening self-efficacy in older adults living in the community.	RCT by clusters	n=262; chronic musculoskeletal pain	Non-pharmacological self-care for pain: adapted light exercises, coping strategies, daily journals, pain education, and sharing experiences in a group setting.	Usual care	SF-12 mental and physical: There were no significant differences between the groups in the scores (p>0.05).	It reduced the intensity of pain and increased self-efficacy for pain management.	Short follow-up, variable engagement, single-center study.

Zhang et al., 2024	To evaluate the impact of personalized health education on elderly patients with chronic diseases in a general practice setting.	Controlled RCT	n=126; hypertension; diabetes	Individualized health education: personalized plan, disease management, self-care support, ongoing follow-up, and family guidance when necessary.	Usual care	SF-36: significant improvement in physical and mental domains ($p<0.05$)	It significantly improved understanding of the disease, health literacy, self-care, psychological well-being, and physical health, and reduced the risk of adverse events.	Small sample size, single-center study, and short follow-up period.
Tey et al., 2019	To determine the effectiveness of the "Living Successfully with Low Vision" (LSLV) self-management program in improving outcomes.	RCT	n=128; macular degeneration, glaucoma, diabetic retinopathy	Strategies for functional management, visual adaptation, problem-solving, emotional support, and activity planning.	Usual care	IVI: was not effective in improving quality of life ($p\geq0.05$)	The intervention improved self-efficacy and the use of coping strategies, but was not sufficient to produce an improvement in quality of life.	High refusal rate, limited to a single treatment institution.

Sun et al., 2023	To investigate the effects of resistance exercise (RE) in elderly cancer patients.	RCT	n=240; cancer undergoing treatment (chemotherapy or radiotherapy)	supervised resistance exercise	Usual care	SF-36: significant improvement in functional capacity, vitality, and overall health (p<0.05)	It reduced fatigue, improved quality of life, physical function, and psychological resilience in older adults.	Blindness impossible; adhesion variable.
Selzler et al., 2021	Comparing how an enhanced pulmonary rehabilitation (APR) program improves self-care, respiratory symptoms, and quality of life compared to traditional rehabilitation.	RCT	n=207; COPD	Self-management and pulmonary rehabilitation: training in breathing techniques, dyspnea management, energy conservation, home exercise plan, symptom	Traditional pulmonary rehabilitation	SGRQ: Both groups showed a significant improvement in quality of life after the intervention (p < 0.05).	The addition of self-management did not increase the clinical benefit in the short term.	Small sample size; subsequent modifications to the protocol.

				monitoring, individualized goals, and problem-solving strategies.				
Saghaee et al., 2020	To evaluate the effectiveness of the Diabetes Self-Management Education Program on self-efficacy , quality of life, self-care activities, depression, and loneliness.	RCT pilot	n=34; type 2 diabetes	Self-care behaviors: monitoring glucose levels, adhering to diets, seeking help from reliable sources.	Usual care	DQoL - BCI: Significant improvement in quality of life in the intervention group compared to the control group (p = 0.04)	The program has a positive effect on quality of life and in the domain of medical control of self-efficacy	Small sample size, pilot study, short follow-up period, and conducted in a single facility.
Rahimi et al., 2020	To assess whether home monitoring with remote specialized support	RCT	n=202; heart failure	Daily home monitoring (weight, blood pressure, symptoms)	Usual care	MLWHF: There was no significant difference between	There was no robust evidence of improvement in the use of evidence-based treatments or	It requires technology and commitment .

	improves treatment and quality of life compared to home monitoring without clinical support.			via tablet with guidance for self-management (recording, tracking, and identifying clinical changes).		the physical, emotional, and social domains ($p \geq 0.63$).	in health-related quality of life.	
Metzner et al., 2023	LoChro care model (collaborative, scalable, and personalized management) improves clinical outcomes and quality of life.	RCT	n = 491; cardiovascular diseases, cancer, diseases , musculoskeletal disorders, diabetes mellitus, dementia and depression	Collaborative management: care planning, coordination between services, individualized goals, and support for ongoing self-management.	Usual care	Quality of life was assessed using the EQ-5D-3L and EQ-VAS. There was no significant difference between the groups over 12 months ($p = 0.44$).	Support for self-care did not produce an improvement in quality of life or symptoms, indicating that exclusively educational/organizational interventions may be insufficient in advanced multimorbidity .	Quality of life assessment using a generic instrument; high clinical complexity reduces the potential for change; the impact of COVID-19 reduced face-to-face interactions;

								adherence is variable.
Mizukawa et al., 2019	To assess whether nurse-led collaborative management with telemonitoring improves quality of life and reduces readmissions.	RCT	n=59; heart failure	telemonitoring with continuous telephone support from the nurse for review and adjustment of the care plan.	Usual care	MLWHFQ: The collaborative management group showed a significant improvement in QoL scores compared to usual care ($p < 0.05$).	It improved quality of life and reduced readmissions , suggesting that continuous nursing support is crucial for the effectiveness of self-care.	Small sample size; high dropout rate; pilot study with limited statistical power.
Wu et al., 2023	To analyze the role of health education in the management of chronic diseases in older adults in the community and the	RCT	n=120; elderly individuals with hypertension, type 2 diabetes mellitus, coronary heart	Community health education model: structured teaching on disease, adherence, nutrition, exercise,	Usual care	QLICD-GM : There was significant improvement in the physical, psychological, and social	It improves understanding of the disease and increases adherence to medical guidelines, while reducing anxiety, depressed mood, and improving quality of life.	Short follow-up period; lack of long-term evaluation; intervention implemented in a single center.

	countermeasures.		disease, heart failure, rheumatoid arthritis, and chronic lumbar spine injuries.	self-management, and remote monitoring of blood pressure and glucose levels.		domains (all $p < 0.05$).		
Benthien et al., 2022	Evaluating the effect of Proactive Health Support (PaHS): telephone-based self-management support for people at risk of hospitalization .	RCT	n = 6,402; diabetes, heart disease, chronic lung disease, osteoarthritis , kidney disease, cancer.	Structured telephone support for self-care conducted by a nurse.	Usual precautions.	SF-36v2: showed significant improvement in the mental health component ($p < 0.0001$).	The PaHS intervention improved health-related quality of life for all participants and reduced hospital admissions only for people with diabetes.	Lack of blinding; low adherence; and imputation of missing data.
Fisher et al., 2020	To evaluate the effectiveness of a self-management	Pragmatic RCT	n = 59 ; chronic cardiovascular,	Community self-management program :	Usual precautions.	SF-12: There was no significant	The program was viable, but it did not improve the quality of life .	Small sample size; variable adherence.

	program compared to usual care in improving self-management, self-efficacy , service utilization, and quality of life.		renal/urogenital, arthritis, gastrointestinal, endocrine, and sensory conditions.	goal setting, symptom management, communication with the team, and system navigation.		improvement in the physical and mental components (p = 0.20)		
Campbell et al., 2023	To evaluate whether a self-care support program, developed with advertising principles, improves cardiovascular outcomes, medication adherence, and quality of life.	Pragmatic RCT	n = 4761; high cardiovascular risk, disease , stroke, chronic kidney disease or heart failure	Messages promoting health and facilitating the transmission of clinical information to the attending physician. primary care and to the pharmacist	Usual precautions.	EQ-5D: no significant improvement (p > 0.05).	The program reduced hospitalizations sensitive to cardiovascular outpatient care (p = 0.009), but did not improve quality of life.	Absence of blinding, losses during follow-up, and limited generalizability to populations with different socioeconomic profiles.
Sturm et al., 2022	To evaluate the effectiveness of a holistic care	RCT by clusters.	n = 297; hypertension, type 2	Structured conversation about	Usual precautions.	SF-12: there was no	The intervention improved emotional well-being in	Results depend on cultural

	program integrated into Primary Care focused on spirituality, self-care, and social support in older adults.		diabetes, ischemic heart disease, heart failure, chronic lung disease, osteoarthritis , depression, and chronic kidney disease.	spirituality; guidance on the use of safe home remedies for symptom management; and encouragement to participate in community social activities.		significant improvement between the groups in the total sample ($p > 0.05$).	participants who identified with spiritual practices.	adherence and alignment; heterogeneity between units.
Singleton et al., 2023	To evaluate the effectiveness, feasibility, and acceptability of a lifestyle-focused text messaging program (EMPOWER-SMS) for improving self-efficacy ,	Parallel RCT	n=160 women with breast cancer (treatment completed)	Text messages (physical activity, healthy eating, social and emotional well-being, adherence to treatment, managemen	Usual care	EORTC QLQ-C30 and ORTC QLQ-BR23: Neither of the QoL domains showed statistical significance ($p>0.05$).	EMPOWER-SMS was considered viable, low-cost, and well-accepted; it did not improve quality of life, but it did slightly improve adherence to endocrine therapy.	Due to COVID-19 restrictions, half of the participants were monitored by telephone. The body composition data were

	quality of life, and mental and physical health.			t of side effects).				self-reported , which limited the statistical power and accuracy.
Sakakibara et al., 2022	To examine the effectiveness of the tele-guidance program. Stroke Coaching in promoting behavioral and lifestyle changes and improving cardiometabolic indicators and quality of life.	Parallel RCT	n=126 stroke survivors (chronic cerebrovascular disease)	Telecoaching : focusing on lifestyle self-care, personalized goals, self-regulation , self-monitoring , educational manual, and action plan.	Memory Training uses the same sessions and frequency , but focuses on memory strategies, without a focus on lifestyle or self-care.	Improvement in SF-36: significant improvement in stroke. Coach ($p = 0.027$) and also a significant difference between groups in favor of the intervention ($p = 0.014$)	Lifestyle monitoring does not improve lifestyle-related behavior, but it does significantly improve health-related quality of life and HbA1c.	Variable adherence to the program; stroke intensity not entirely homogeneous.

Solis et al., 2024	To evaluate the clinical effectiveness of the PPEP4All-PDD self-care program compared to usual care in people with persistent depressive disorder and their partners/caregivers.	Pragmatic RCT	n=70 patients with persistent depression	Stress management, recognizing patterns of depression, social skills, crisis prevention, coping strategies, and caregiver involvement.	Standard therapy	Self-Rated Happiness (SRH) and Brief Resilience Scale (BRS). There was no significant improvement between groups ($p > 0.05$).	The intervention was no more advanced than usual care; it may have helped prevent further deterioration, but without measurable improvement.	Sample size smaller than planned, losses to follow-up, impact of COVID-19 on the intervention design, medication variations, and lack of a standardized quality of life measure.
Zhang et al., 2021	To evaluate the effect of nursing care based on Roy's Adaptation Model in elderly patients with hypertension.	RCT	n=120 elderly people with hypertension	Roy's model: education for self-care (diet, exercise, proper medication use), blood pressure monitoring,	Usual care	SF-36: significant improvement in most domains and in the total score ($p < 0.05$), except for general health	Roy's Adaptation Model improved self-care, blood pressure control, and quality of life in older adults with hypertension.	self-reported data limiting generalizability.

				emotional support, and family involvement .		status (p = 0.077).		
Wang et al., 2021	To evaluate the effectiveness of family-based and organizational-based healthcare management in nursing for elderly diabetic patients.	RCT	n = 126 ; type 2 diabetes mellitus .	Self-care: family involvement , regular monitoring, home visits, dietary guidance, exercise, medication use, and ongoing follow-up.	Usual care (periodic lectures)	SF-36 : significantly improved in the intervention group in all domains analyzed ($p < 0.001$) compared to the control group.	The intervention improved quality of life, psychological state, and glycemic control when compared to usual care.	Single center, short follow-up, self-reported measures
Wong & Wong, 2020	To evaluate the impact of a proactive nurse-led self-care program on psychological	RCT	n = 457 ; hypertension, diabetes, heart disease, osteoarticular	Self-care program: home visits; follow-up calls; goal setting; problem	Usual care	SF-12 (Mental Component – MCS): similar improvement in both	The program improved emotional well-being and reduced depressive symptoms over time, but did not improve mental	Sample of independent older adults, short follow-up period, and study in a

	health and the mental component of quality of life.			solving; self-care monitoring.		groups; no difference between groups ($p > 0.05$).	quality of life beyond usual care.	single urban setting.
McGowan & Hofer , 2022	To assess whether peer coaching , with or without the use of home electronic devices, improves self-care and self-perceived health outcomes.	RCT	n = 163 ; arthritis, cardiovascular disease, chronic pain, neurological disorders, respiratory disease, cancer	Weekly telephone coaching: self-management, goal setting, and habit maintenance .	Control: educational manual.	SF-36: Coach : improvement in energy/fatigue and social functioning ($p < 0.05$). Coach + Devices : additional improvement in depression ($p = 0.003$), self-efficacy ($p = 0.002$), pain ($p < 0.01$) and	Peer coaching improved components of QoL and emotional well-being. The addition of devices amplified the effects, especially for self -efficacy , energy, well-being , and pain. The model can complement primary care.	Higher dropout rate in the Coach+Devices group (difficulty using the devices). Study conducted in a highly educated population, which reduces generalizability. COVID-19 partially interfered with the data

						general health ($p < 0.05$).		collection process.
Benthien et al., 2022	To evaluate the effect of *Proactive Health Support* in people at high risk of hospitalization.	RCT	n=6402	Structured telephone support for self-care; focused on: knowledge, coping , strategies, self-care, health navigation.	Standard care provided by the public healthcare system.	SF-36v2 – MHCS: significant improvement at 6 months (Est. = 1.50, $p < 0.0001$). Significant improvement also at 3 and 12 months in all 10 scales. Better effects in people with diabetes (MHCS Est. = 2.39; 95% CI).	The PaHS improved quality of life across all scales assessed, without reducing hospitalizations in the overall sample, but showed clinically relevant benefits for people with diabetes.	Lack of blinding of participants; possibility of optimism bias; high initial non-participation rate; regional variations in implementation; small effects.

Table S2: Complete search strategies used for each database (Scopus, Web of Science, and EB-SCOhost)

Table S2. Final search strategies and records retrieved

Database	Final search strategy
Scopus (Elsevier)	TITLE-ABS-KEY ((elder* OR "older adult*" OR "aged people" OR senior*) AND ("chronic disease*" OR "chronic illness*" OR "chronic condition*" OR "noncommunicable disease*" OR "chronically ill" OR "long-term conditions") AND ("self-care" OR "self-management" OR "self-efficacy" OR "self-regulation" OR "health behaviors" OR autonomy OR "self-care capacity" OR "self-care behaviors" OR "self-care skills" OR "health education" OR "patient education" OR empowerment OR "management of signs and symptoms" OR "self-management of chronic disease") AND ("quality of life" OR "health outcomes" OR "well-being" OR "health-related quality of life" OR "patient-reported outcomes" OR "clinical outcomes" OR "health status"))
Web of Science – Core Collection (Clarivate Analytics)	ALL=(elder* OR "older adult*" OR "aged people" OR senior*) AND ("chronic disease*" OR "chronic illness" OR "chronic condition" OR "noncommunicable disease" OR "chronically ill" OR "long-term conditions") AND ("self-care" OR "self-management" OR "self-efficacy" OR "self-regulation" OR "health behaviors" OR autonomy OR "self-care capacity" OR "self-care behaviors" OR "self-care skills" OR "health education" OR "patient education" OR empowerment OR "management of signs and symptoms" OR "self-management of chronic disease") AND ("quality of life" OR "health outcomes" OR "well-being" OR "health-related quality of life" OR "patient-reported outcomes" OR "clinical outcomes" OR "health status")
EBSCOhost (all databases, including CINAHL)	TI((elder* OR "older adult*" OR "aged people" OR senior*) AND ("chronic disease*" OR "chronic illness" OR "chronic condition" OR "noncommunicable disease" OR "chronically ill" OR "long-term conditions") AND ("self-care" OR "self-management" OR "self-efficacy" OR "self-regulation" OR "health behaviors" OR autonomy OR "self-care capacity" OR "self-care behaviors" OR "self-care skills" OR "health education" OR "patient education" OR empowerment OR "management of signs and symptoms" OR "self-management of chronic disease") AND ("quality of life" OR "health outcomes" OR "well-being" OR "health-related quality of life" OR "patient-reported outcomes"

	OR "clinical outcomes" OR "health status")) OR AB((elder* OR "older adult*" OR "aged people" OR senior*) AND ("chronic disease*" OR "chronic illness" OR "chronic condition" OR "noncommunicable disease" OR "chronically ill" OR "long-term conditions") AND ("self-care" OR "self-management" OR "self-efficacy" OR "self-regulation" OR "health behaviors" OR autonomy OR "self-care capacity" OR "self-care behaviors" OR "self-care skills" OR "health education" OR "patient education" OR empowerment OR "management of signs and symptoms" OR "self-management of chronic disease") AND ("quality of life" OR "health outcomes" OR "well-being" OR "health-related quality of life" OR "patient-reported outcomes" OR "clinical outcomes" OR "health status")) OR SU((elder* OR "older adult*" OR "aged people" OR senior*) AND ("chronic disease*" OR "chronic illness" OR "chronic condition" OR "noncommunicable disease" OR "chronically ill" OR "long-term conditions") AND ("self-care" OR "self-management" OR "self-efficacy" OR "self-regulation" OR "health behaviors" OR autonomy OR "self-care capacity" OR "self-care behaviors" OR "self-care skills" OR "health education" OR "patient education" OR empowerment OR "management of signs and symptoms" OR "self-management of chronic disease") AND ("quality of life" OR "health outcomes" OR "well-being" OR "health-related quality of life" OR "patient-reported outcomes" OR "clinical outcomes" OR "health status"))

Table S3: Keywords and controlled vocabulary terms used in the search strategy

Table S3. Terms used in the search strategy.

#1	Elder* [Title/Abstract]
#2	"older adult*" [Title/Abstract]
#3	"aged people" [Title/Abstract]
#5	senior* [Title/Abstract]
#6	#1 OR #2 OR #3 OR #4
#7	"Chronic Illness" [Title/Abstract]
#8	"Chronic Condition" [Title/Abstract]
#9	"Noncommunicable disease" [Title/Abstract]
#10	"Chronically ill" [Title/Abstract]
#11	"long-term conditions" [Title/Abstract]
#12	#6 OR #7 OR #8 OR #9 OR #10 OR #11
#13	"Self-care" [Title/Abstract]
#14	"self-management" [Title/Abstract]
#15	"self-efficacy" [Title/Abstract]
#16	"self-regulation" [Title/Abstract]
#17	"health behaviors" [Título/Abstract]
#18	"autonomy" [Title/Abstract]
#19	"self-care capacity" [Title/Abstract]
#20	"self-care behaviors" [Title/Abstract]
#21	"self-care skills" [Title/Abstract]
#22	"health education" [Title/Abstract]
#23	"patient education" [Title/Abstract]
#24	"empowerment" [Title/Abstract]
#25	"management of signs and symptoms" [Title/Abstract]
#26	"self-management of chronic disease" [Title/Abstract]
#27	#13 OR #14 OR #15 OR #16 OR #17 OR #18 OR #19 OR #20 OR #21 OR #22 OR #23 OR #24 OR #25 OR #26
#28	"Quality of life" [Title/Abstract]
#29	"health outcomes" [Title/Abstract]
#30	"well-being" [Title/Abstract]
#31	"health-related quality of life" [Title/Abstract]
#32	"patient-reported outcomes" [Title/Abstract]
#33	"clinical outcomes" [Title/Abstract]
#34	"health status" [Title/Abstract]
#35	#28 OR #29 OR #30 OR #31 OR #32 OR #33 OR #34
#36	#5 AND #12 AND #27 AND #35

JBI CRITICAL APPRAISAL CHECKLIST FOR RANDOMIZED CONTROLLED TRIALS

Reviewer ______________________________________Date: 01/05/2025

Author **Mizukawa et al, 2019**_________________________Year__________ Record Number__________

	Yes	No	Unclear	NA
1. Was true randomization used for assignment of participants to treatment groups?	■	☐	☐	☐
2. Was allocation to treatment groups concealed?	☐	☐	■	☐
3. Were treatment groups similar at the baseline?	■	☐	☐	☐
4. Were participants blind to treatment assignment?	☐	■	☐	☐
5. Were those delivering treatment blind to treatment assignment?	☐	■	☐	☐
6. Were outcomes assessors blind to treatment assignment?	☐	☐	■	☐
7. Were treatment groups treated identically other than the intervention of interest?	■	☐	☐	☐
8. Was follow up complete and if not, were differences between groups in terms of their follow up adequately described and analyzed?	■	☐	☐	☐
9. Were participants analyzed in the groups to which they were randomized?	■	☐	☐	☐
10. Were outcomes measured in the same way for treatment groups?	■	☐	☐	☐
11. Were outcomes measured in a reliable way?	■	☐	☐	☐
12. Was appropriate statistical analysis used?	■	☐	☐	☐
13. Was the trial design appropriate, and any deviations from the standard RCT design (individual randomization, parallel groups) accounted for in the conduct and analysis of the trial?	■	☐	☐	☐

Overall appraisal: Include ■ Exclude ☐ Seek further info ☐

Comments (Including reason for exclusion)

© JBI, 2020. All rights reserved. JBI grants use of these tools for research purposes only. All other enquiries should be sent to jbisynthesis@adelaide.edu.au.

© JBI, 2020. All rights reserved. JBI grants use of these tools for research purposes only. All other enquiries should be sent to jbisynthesis@adelaide.edu.au.

JBI CRITICAL APPRAISAL CHECKLIST FOR RANDOMIZED CONTROLLED TRIALS

Reviewer____________________________________Date______________________________

Author **Miklavcic et al., 2020**______________________Year_________ Record Number_________

	Yes	No	Unclear	NA
1. Was true randomization used for assignment of participants to treatment groups?	■	☐	☐	☐
2. Was allocation to treatment groups concealed?	☐	☐	■	☐
3. Were treatment groups similar at the baseline?	■	☐	☐	☐
4. Were participants blind to treatment assignment?	☐	■	☐	☐
5. Were those delivering treatment blind to treatment assignment?	☐	■	☐	☐
6. Were outcomes assessors blind to treatment assignment?	☐	☐	■	☐
7. Were treatment groups treated identically other than the intervention of interest?	■	☐	☐	☐
8. Was follow up complete and if not, were differences between groups in terms of their follow up adequately described and analyzed?	■	☐	☐	☐
9. Were participants analyzed in the groups to which they were randomized?	■	☐	☐	☐
10. Were outcomes measured in the same way for treatment groups?	■	☐	☐	☐
11. Were outcomes measured in a reliable way?	■	☐	☐	☐
12. Was appropriate statistical analysis used?	■	☐	☐	☐
13. Was the trial design appropriate, and any deviations from the standard RCT design (individual randomization, parallel groups) accounted for in the conduct and analysis of the trial?	■	☐	☐	☐

Overall appraisal: Include ■ Exclude ☐ Seek further info ☐

Comments (Including reason for exclusion)

____

____

____

© JBI, 2020. All rights reserved. JBI grants use of these tools for research purposes only. All other enquiries should be sent to jbisynthesis@adelaide.edu.au.

JBI CRITICAL APPRAISAL CHECKLIST FOR RANDOMIZED CONTROLLED TRIALS

Reviewer____Date_________________________________

Author **Metzner et al., 2023**____________________________Year__________ Record Number__________

	Yes	No	Unclear	NA
1. Was true randomization used for assignment of participants to treatment groups?	■	☐	☐	☐
2. Was allocation to treatment groups concealed?	☐	☐	■	☐
3. Were treatment groups similar at the baseline?	■	☐	☐	☐
4. Were participants blind to treatment assignment?	☐	■	☐	☐
5. Were those delivering treatment blind to treatment assignment?	☐	■	☐	☐
6. Were outcomes assessors blind to treatment assignment?	☐	☐	■	☐
7. Were treatment groups treated identically other than the intervention of interest?	■	☐	☐	☐
8. Was follow up complete and if not, were differences between groups in terms of their follow up adequately described and analyzed?	■	☐	☐	☐
9. Were participants analyzed in the groups to which they were randomized?	■	☐	☐	☐
10. Were outcomes measured in the same way for treatment groups?	■	☐	☐	☐
11. Were outcomes measured in a reliable way?	■	☐	☐	☐
12. Was appropriate statistical analysis used?	■	☐	☐	☐
13. Was the trial design appropriate, and any deviations from the standard RCT design (individual randomization, parallel groups) accounted for in the conduct and analysis of the trial?	■	☐	☐	☐

Overall appraisal: Include ■ Exclude ☐ Seek further info ☐

Comments (Including reason for exclusion)

____

____

____

© JBI, 2020. All rights reserved. JBI grants use of these tools for research purposes only. All other enquiries should be sent to jbisynthesis@adelaide.edu.au.

JBI CRITICAL APPRAISAL CHECKLIST FOR RANDOMIZED CONTROLLED TRIALS

Reviewer______________________________________Date_______________________________

Author **McGowan & Hofer, 2022**____________________Year_________ Record Number_________

	Yes	No	Unclear	NA
1. Was true randomization used for assignment of participants to treatment groups?	■	☐	☐	☐
2. Was allocation to treatment groups concealed?	☐	■	☐	☐
3. Were treatment groups similar at the baseline?	■	☐	☐	☐
4. Were participants blind to treatment assignment?	☐	■	☐	☐
5. Were those delivering treatment blind to treatment assignment?	☐	■	☐	☐
6. Were outcomes assessors blind to treatment assignment?	☐	■	☐	☐
7. Were treatment groups treated identically other than the intervention of interest?	■	☐	☐	☐
8. Was follow up complete and if not, were differences between groups in terms of their follow up adequately described and analyzed?	■	☐	☐	☐
9. Were participants analyzed in the groups to which they were randomized?	■	☐	☐	☐
10. Were outcomes measured in the same way for treatment groups?	■	☐	☐	☐
11. Were outcomes measured in a reliable way?	■	☐	☐	☐
12. Was appropriate statistical analysis used?	■	☐	☐	☐
13. Was the trial design appropriate, and any deviations from the standard RCT design (individual randomization, parallel groups) accounted for in the conduct and analysis of the trial?	■	☐	☐	☐

Overall appraisal: Include ■ Exclude ☐ Seek further info ☐

Comments (Including reason for exclusion)

____

____

© JBI, 2020. All rights reserved. JBI grants use of these tools for research purposes only. All other enquiries should be sent to jbisynthesis@adelaide.edu.au.

JBI CRITICAL APPRAISAL CHECKLIST FOR RANDOMIZED CONTROLLED TRIALS

Reviewer______________________________________Date_______________________________

Author **Markle-Reid et al., 2021**____________________Year__________ Record Number__________

	Yes	No	Unclear	NA
1. Was true randomization used for assignment of participants to treatment groups?	■	☐	☐	☐
2. Was allocation to treatment groups concealed?	■	☐	☐	☐
3. Were treatment groups similar at the baseline?	■	☐	☐	☐
4. Were participants blind to treatment assignment?	☐	■	☐	☐
5. Were those delivering treatment blind to treatment assignment?	☐	■	☐	☐
6. Were outcomes assessors blind to treatment assignment?	■	☐	☐	☐
7. Were treatment groups treated identically other than the intervention of interest?	■	☐	☐	☐
8. Was follow up complete and if not, were differences between groups in terms of their follow up adequately described and analyzed?	■	☐	☐	☐
9. Were participants analyzed in the groups to which they were randomized?	■	☐	☐	☐
10. Were outcomes measured in the same way for treatment groups?	■	☐	☐	☐
11. Were outcomes measured in a reliable way?	■	☐	☐	☐
12. Was appropriate statistical analysis used?	■	☐	☐	☐
13. Was the trial design appropriate, and any deviations from the standard RCT design (individual randomization, parallel groups) accounted for in the conduct and analysis of the trial?	■	☐	☐	☐

Overall appraisal: Include ■ Exclude ☐ Seek further info ☐

Comments (Including reason for exclusion)

____

____

© JBI, 2020. All rights reserved. JBI grants use of these tools for research purposes only. All other enquiries should be sent to jbisynthesis@adelaide.edu.au.

JBI CRITICAL APPRAISAL CHECKLIST FOR RANDOMIZED CONTROLLED TRIALS

Reviewer______________________________________Date________________________________

Author **Liu et al., 2021**____________________________Year__________ Record Number__________

	Yes	No	Unclear	NA
1. Was true randomization used for assignment of participants to treatment groups?	■	☐	☐	☐
2. Was allocation to treatment groups concealed?	☐	☐	■	☐
3. Were treatment groups similar at the baseline?	■	☐	☐	☐
4. Were participants blind to treatment assignment?	☐	■	☐	☐
5. Were those delivering treatment blind to treatment assignment?	☐	■	☐	☐
6. Were outcomes assessors blind to treatment assignment?	☐	☐	■	☐
7. Were treatment groups treated identically other than the intervention of interest?	■	☐	☐	☐
8. Was follow up complete and if not, were differences between groups in terms of their follow up adequately described and analyzed?	■	☐	☐	☐
9. Were participants analyzed in the groups to which they were randomized?	■	☐	☐	☐
10. Were outcomes measured in the same way for treatment groups?	■	☐	☐	☐
11. Were outcomes measured in a reliable way?	■	☐	☐	☐
12. Was appropriate statistical analysis used?	■	☐	☐	☐
13. Was the trial design appropriate, and any deviations from the standard RCT design (individual randomization, parallel groups) accounted for in the conduct and analysis of the trial?	■	☐	☐	☐

Overall appraisal: Include ■ Exclude ☐ Seek further info ☐

Comments (Including reason for exclusion)

____

____

© JBI, 2020. All rights reserved. JBI grants use of these tools for research purposes only. All other enquiries should be sent to jbisynthesis@adelaide.edu.au.

JBI CRITICAL APPRAISAL CHECKLIST FOR RANDOMIZED CONTROLLED TRIALS

Reviewer_______________________________________Date________________________________

Author **Lipsitz et al., 2019**__________________________Year__________ Record Number__________

	Yes	No	Unclear	NA
1. Was true randomization used for assignment of participants to treatment groups?	■	☐	☐	☐
2. Was allocation to treatment groups concealed?	☐	☐	■	☐
3. Were treatment groups similar at the baseline?	■	☐	☐	☐
4. Were participants blind to treatment assignment?	☐	■	☐	☐
5. Were those delivering treatment blind to treatment assignment?	☐	■	☐	☐
6. Were outcomes assessors blind to treatment assignment?	■	☐	☐	☐
7. Were treatment groups treated identically other than the intervention of interest?	■	☐	☐	☐
8. Was follow up complete and if not, were differences between groups in terms of their follow up adequately described and analyzed?	■	☐	☐	☐
9. Were participants analyzed in the groups to which they were randomized?	■	☐	☐	☐
10. Were outcomes measured in the same way for treatment groups?	■	☐	☐	☐
11. Were outcomes measured in a reliable way?	■	☐	☐	☐
12. Was appropriate statistical analysis used?	■	☐	☐	☐
13. Was the trial design appropriate, and any deviations from the standard RCT design (individual randomization, parallel groups) accounted for in the conduct and analysis of the trial?	■	☐	☐	☐

Overall appraisal: Include ■ Exclude ☐ Seek further info ☐

Comments (Including reason for exclusion)

____

____

© JBI, 2020. All rights reserved. JBI grants use of these tools for research purposes only. All other enquiries should be sent to jbisynthesis@adelaide.edu.au.

JBI CRITICAL APPRAISAL CHECKLIST FOR RANDOMIZED CONTROLLED TRIALS

Reviewer_______________________________________Date________________________________

Author **Fisher et al., 2020**__________________________Year__________ Record Number__________

	Yes	No	Unclear	NA
1. Was true randomization used for assignment of participants to treatment groups?	■	☐	☐	☐
2. Was allocation to treatment groups concealed?	☐	■	☐	☐
3. Were treatment groups similar at the baseline?	■	☐	☐	☐
4. Were participants blind to treatment assignment?	☐	■	☐	☐
5. Were those delivering treatment blind to treatment assignment?	☐	■	☐	☐
6. Were outcomes assessors blind to treatment assignment?	☐	■	☐	☐
7. Were treatment groups treated identically other than the intervention of interest?	■	☐	☐	☐
8. Was follow up complete and if not, were differences between groups in terms of their follow up adequately described and analyzed?	■	☐	☐	☐
9. Were participants analyzed in the groups to which they were randomized?	■	☐	☐	☐
10. Were outcomes measured in the same way for treatment groups?	■	☐	☐	☐
11. Were outcomes measured in a reliable way?	■	☐	☐	☐
12. Was appropriate statistical analysis used?	■	☐	☐	☐
13. Was the trial design appropriate, and any deviations from the standard RCT design (individual randomization, parallel groups) accounted for in the conduct and analysis of the trial?	■	☐	☐	☐

Overall appraisal: Include ■ Exclude ☐ Seek further info ☐

Comments (Including reason for exclusion)

____

____

© JBI, 2020. All rights reserved. JBI grants use of these tools for research purposes only. All other enquiries should be sent to jbisynthesis@adelaide.edu.au.

JBI CRITICAL APPRAISAL CHECKLIST FOR RANDOMIZED CONTROLLED TRIALS

Reviewer_______________________________________Date________________________________

Author **Campbell et al., 2023**________________________Year__________ Record Number__________

	Yes	No	Unclear	NA
1. Was true randomization used for assignment of participants to treatment groups?	☒	☐	☐	☐
2. Was allocation to treatment groups concealed?	☒	☐	☐	☐
3. Were treatment groups similar at the baseline?	☐	☒	☐	☐
4. Were participants blind to treatment assignment?	☐	☒	☐	☐
5. Were those delivering treatment blind to treatment assignment?	☒	☐	☐	☐
6. Were outcomes assessors blind to treatment assignment?	☒	☐	☐	☐
7. Were treatment groups treated identically other than the intervention of interest?	☒	☐	☐	☐
8. Was follow up complete and if not, were differences between groups in terms of their follow up adequately described and analyzed?	☒	☐	☐	☐
9. Were participants analyzed in the groups to which they were randomized?	☒	☐	☐	☐
10. Were outcomes measured in the same way for treatment groups?	☒	☐	☐	☐
11. Were outcomes measured in a reliable way?	☒	☐	☐	☐
12. Was appropriate statistical analysis used?	☒	☐	☐	☐
13. Was the trial design appropriate, and any deviations from the standard RCT design (individual randomization, parallel groups) accounted for in the conduct and analysis of the trial?	☒	☐	☐	☐

Overall appraisal: Include ☒ Exclude ☐ Seek further info ☐

Comments (Including reason for exclusion)

____

____

© JBI, 2020. All rights reserved. JBI grants use of these tools for research purposes only. All other enquiries should be sent to jbisynthesis@adelaide.edu.au.

JBI CRITICAL APPRAISAL CHECKLIST FOR RANDOMIZED CONTROLLED TRIALS

Reviewer______________________________________Date_______________________________

Author **Benthien et al., 2022**_______________________Year__________ Record Number__________

	Yes	No	Unclear	NA
1. Was true randomization used for assignment of participants to treatment groups?	■	☐	☐	☐
2. Was allocation to treatment groups concealed?	■	☐	☐	☐
3. Were treatment groups similar at the baseline?	■	☐	☐	☐
4. Were participants blind to treatment assignment?	☐	■	☐	☐
5. Were those delivering treatment blind to treatment assignment?	☐	■	☐	☐
6. Were outcomes assessors blind to treatment assignment?	■	☐	☐	☐
7. Were treatment groups treated identically other than the intervention of interest?	■	☐	☐	☐
8. Was follow up complete and if not, were differences between groups in terms of their follow up adequately described and analyzed?	■	☐	☐	☐
9. Were participants analyzed in the groups to which they were randomized?	■	☐	☐	☐
10. Were outcomes measured in the same way for treatment groups?	■	☐	☐	☐
11. Were outcomes measured in a reliable way?	■	☐	☐	☐
12. Was appropriate statistical analysis used?	■	☐	☐	☐
13. Was the trial design appropriate, and any deviations from the standard RCT design (individual randomization, parallel groups) accounted for in the conduct and analysis of the trial?	■	☐	☐	☐

Overall appraisal: Include ■ Exclude ☐ Seek further info ☐

Comments (Including reason for exclusion)

____

____

© JBI, 2020. All rights reserved. JBI grants use of these tools for research purposes only. All other enquiries should be sent to jbisynthesis@adelaide.edu.au.

JBI CRITICAL APPRAISAL CHECKLIST FOR RANDOMIZED CONTROLLED TRIALS

Reviewer______________________________________Date_______________________________

Author Zhang et al., 2024________________________Year_________ Record Number_________

	Yes	No	Unclear	NA
1. Was true randomization used for assignment of participants to treatment groups?	■	☐	☐	☐
2. Was allocation to treatment groups concealed?	☐	☐	■	☐
3. Were treatment groups similar at the baseline?	■	☐	☐	☐
4. Were participants blind to treatment assignment?	☐	■	☐	☐
5. Were those delivering treatment blind to treatment assignment?	☐	■	☐	☐
6. Were outcomes assessors blind to treatment assignment?	☐	☐	■	☐
7. Were treatment groups treated identically other than the intervention of interest?	■	☐	☐	☐
8. Was follow up complete and if not, were differences between groups in terms of their follow up adequately described and analyzed?	■	☐	☐	☐
9. Were participants analyzed in the groups to which they were randomized?	■	☐	☐	☐
10. Were outcomes measured in the same way for treatment groups?	■	☐	☐	☐
11. Were outcomes measured in a reliable way?	■	☐	☐	☐
12. Was appropriate statistical analysis used?	■	☐	☐	☐
13. Was the trial design appropriate, and any deviations from the standard RCT design (individual randomization, parallel groups) accounted for in the conduct and analysis of the trial?	■	☐	☐	☐

Overall appraisal: Include ■ Exclude ☐ Seek further info ☐

Comments (Including reason for exclusion)

____

____

© JBI, 2020. All rights reserved. JBI grants use of these tools for research purposes only. All other enquiries should be sent to jbisynthesis@adelaide.edu.au.

JBI CRITICAL APPRAISAL CHECKLIST FOR RANDOMIZED CONTROLLED TRIALS

Reviewer_______________________________________Date________________________________

Author: Zhang et al, 2021_________________________Year__________ Record Number__________

	Yes	No	Unclear	NA
1. Was true randomization used for assignment of participants to treatment groups?	■	☐	☐	☐
2. Was allocation to treatment groups concealed?	☐	☐	■	☐
3. Were treatment groups similar at the baseline?	■	☐	☐	☐
4. Were participants blind to treatment assignment?	☐	■	☐	☐
5. Were those delivering treatment blind to treatment assignment?	☐	■	☐	☐
6. Were outcomes assessors blind to treatment assignment?	☐	☐	■	☐
7. Were treatment groups treated identically other than the intervention of interest?	■	☐	☐	☐
8. Was follow up complete and if not, were differences between groups in terms of their follow up adequately described and analyzed?	■	☐	☐	☐
9. Were participants analyzed in the groups to which they were randomized?	■	☐	☐	☐
10. Were outcomes measured in the same way for treatment groups?	■	☐	☐	☐
11. Were outcomes measured in a reliable way?	■	☐	☐	☐
12. Was appropriate statistical analysis used?	■	☐	☐	☐
13. Was the trial design appropriate, and any deviations from the standard RCT design (individual randomization, parallel groups) accounted for in the conduct and analysis of the trial?	■	☐	☐	☐

Overall appraisal: Include ■ Exclude ☐ Seek further info ☐

Comments (Including reason for exclusion)

____

____

© JBI, 2020. All rights reserved. JBI grants use of these tools for research purposes only. All other enquiries should be sent to jbisynthesis@adelaide.edu.au.

JBI CRITICAL APPRAISAL CHECKLIST FOR RANDOMIZED CONTROLLED TRIALS

Reviewer______________________________________Date_______________________________

Author Yang, J., Hu, H. L., Li, Y. M., 2020_____________Year__________ Record Number__________

	Yes	No	Unclear	NA
1. Was true randomization used for assignment of participants to treatment groups?	■	☐	☐	☐
2. Was allocation to treatment groups concealed?	☐	☐	■	☐
3. Were treatment groups similar at the baseline?	■	☐	☐	☐
4. Were participants blind to treatment assignment?	☐	■	☐	☐
5. Were those delivering treatment blind to treatment assignment?	☐	■	☐	☐
6. Were outcomes assessors blind to treatment assignment?	☐	■	☐	☐
7. Were treatment groups treated identically other than the intervention of interest?	■	☐	☐	☐
8. Was follow up complete and if not, were differences between groups in terms of their follow up adequately described and analyzed?	☐	☐	■	☐
9. Were participants analyzed in the groups to which they were randomized?	■	☐	☐	☐
10. Were outcomes measured in the same way for treatment groups?	■	☐	☐	☐
11. Were outcomes measured in a reliable way?	■	☐	☐	☐
12. Was appropriate statistical analysis used?	■	☐	☐	☐
13. Was the trial design appropriate, and any deviations from the standard RCT design (individual randomization, parallel groups) accounted for in the conduct and analysis of the trial?	■	☐	☐	☐

Overall appraisal: Include ■ Exclude ☐ Seek further info ☐

Comments (Including reason for exclusion)

____

____

© JBI, 2020. All rights reserved. JBI grants use of these tools for research purposes only. All other enquiries should be sent to jbisynthesis@adelaide.edu.au.

JBI CRITICAL APPRAISAL CHECKLIST FOR RANDOMIZED CONTROLLED TRIALS

Reviewer______________________________________Date_______________________________

Author Wu, H. Z., Lin, W. Y., Li, Y. K., 2023____________Year_________ Record Number_________

	Yes	No	Unclear	NA
1. Was true randomization used for assignment of participants to treatment groups?	■	☐	☐	☐
2. Was allocation to treatment groups concealed?	☐	☐	■	☐
3. Were treatment groups similar at the baseline?	■	☐	☐	☐
4. Were participants blind to treatment assignment?	☐	■	☐	☐
5. Were those delivering treatment blind to treatment assignment?	☐	☐	■	☐
6. Were outcomes assessors blind to treatment assignment?	☐	☐	■	☐
7. Were treatment groups treated identically other than the intervention of interest?	■	☐	☐	☐
8. Was follow up complete and if not, were differences between groups in terms of their follow up adequately described and analyzed?	■	☐	☐	☐
9. Were participants analyzed in the groups to which they were randomized?	■	☐	☐	☐
10. Were outcomes measured in the same way for treatment groups?	■	☐	☐	☐
11. Were outcomes measured in a reliable way?	■	☐	☐	☐
12. Was appropriate statistical analysis used?	■	☐	☐	☐
13. Was the trial design appropriate, and any deviations from the standard RCT design (individual randomization, parallel groups) accounted for in the conduct and analysis of the trial?	■	☐	☐	☐

Overall appraisal: Include ■ Exclude ☐ Seek further info ☐

Comments (Including reason for exclusion)

____

____

© JBI, 2020. All rights reserved. JBI grants use of these tools for research purposes only. All other enquiries should be sent to jbisynthesis@adelaide.edu.au.

JBI CRITICAL APPRAISAL CHECKLIST FOR RANDOMIZED CONTROLLED TRIALS

Reviewer_______________________________________Date_______________________________

Author Wong et al, 2022_________________________Year__________ Record Number__________

	Yes	No	Unclear	NA
1. Was true randomization used for assignment of participants to treatment groups?	■	☐	☐	☐
2. Was allocation to treatment groups concealed?	■	☐	☐	☐
3. Were treatment groups similar at the baseline?	■	☐	☐	☐
4. Were participants blind to treatment assignment?	☐	■	☐	☐
5. Were those delivering treatment blind to treatment assignment?	☐	■	☐	☐
6. Were outcomes assessors blind to treatment assignment?	■	☐	☐	☐
7. Were treatment groups treated identically other than the intervention of interest?	■	☐	☐	☐
8. Was follow up complete and if not, were differences between groups in terms of their follow up adequately described and analyzed?	■	☐	☐	☐
9. Were participants analyzed in the groups to which they were randomized?	■	☐	☐	☐
10. Were outcomes measured in the same way for treatment groups?	■	☐	☐	☐
11. Were outcomes measured in a reliable way?	■	☐	☐	☐
12. Was appropriate statistical analysis used?	■	☐	☐	☐
13. Was the trial design appropriate, and any deviations from the standard RCT design (individual randomization, parallel groups) accounted for in the conduct and analysis of the trial?	■	☐	☐	☐

Overall appraisal: Include ■ Exclude ☐ Seek further info ☐

Comments (Including reason for exclusion)

____

____

© JBI, 2020. All rights reserved. JBI grants use of these tools for research purposes only. All other enquiries should be sent to jbisynthesis@adelaide.edu.au.

JBI CRITICAL APPRAISAL CHECKLIST FOR RANDOMIZED CONTROLLED TRIALS

Reviewer____Date_________________________________

Author Wong & Wong, 2020________________________Year__________ Record Number__________

	Yes	No	Unclear	NA
1. Was true randomization used for assignment of participants to treatment groups?	■	☐	☐	☐
2. Was allocation to treatment groups concealed?	■	☐	☐	☐
3. Were treatment groups similar at the baseline?	■	☐	☐	☐
4. Were participants blind to treatment assignment?	☐	■	☐	☐
5. Were those delivering treatment blind to treatment assignment?	☐	■	☐	☐
6. Were outcomes assessors blind to treatment assignment?	■	☐	☐	☐
7. Were treatment groups treated identically other than the intervention of interest?	■	☐	☐	☐
8. Was follow up complete and if not, were differences between groups in terms of their follow up adequately described and analyzed?	■	☐	☐	☐
9. Were participants analyzed in the groups to which they were randomized?	■	☐	☐	☐
10. Were outcomes measured in the same way for treatment groups?	■	☐	☐	☐
11. Were outcomes measured in a reliable way?	■	☐	☐	☐
12. Was appropriate statistical analysis used?	■	☐	☐	☐
13. Was the trial design appropriate, and any deviations from the standard RCT design (individual randomization, parallel groups) accounted for in the conduct and analysis of the trial?	■	☐	☐	☐

Overall appraisal: Include ■ Exclude ☐ Seek further info ☐

Comments (Including reason for exclusion)

____

____

© JBI, 2020. All rights reserved. JBI grants use of these tools for research purposes only. All other enquiries should be sent to jbisynthesis@adelaide.edu.au.

JBI CRITICAL APPRAISAL CHECKLIST FOR RANDOMIZED CONTROLLED TRIALS

Reviewer____Date________________________________

Author Wang et al., 2020 Year____________ Record Number________________________

	Yes	No	Unclear	NA
1. Was true randomization used for assignment of participants to treatment groups?	■	☐	☐	☐
2. Was allocation to treatment groups concealed?	☐	☐	■	☐
3. Were treatment groups similar at the baseline?	■	☐	☐	☐
4. Were participants blind to treatment assignment?	☐	■	☐	☐
5. Were those delivering treatment blind to treatment assignment?	☐	■	☐	☐
6. Were outcomes assessors blind to treatment assignment?	☐	☐	■	☐
7. Were treatment groups treated identically other than the intervention of interest?	■	☐	☐	☐
8. Was follow up complete and if not, were differences between groups in terms of their follow up adequately described and analyzed?	■	☐	☐	☐
9. Were participants analyzed in the groups to which they were randomized?	■	☐	☐	☐
10. Were outcomes measured in the same way for treatment groups?	■	☐	☐	☐
11. Were outcomes measured in a reliable way?	■	☐	☐	☐
12. Was appropriate statistical analysis used?	■	☐	☐	☐
13. Was the trial design appropriate, and any deviations from the standard RCT design (individual randomization, parallel groups) accounted for in the conduct and analysis of the trial?	■	☐	☐	☐

Overall appraisal: Include ☐ Exclude ☐ Seek further info ☐

Comments (Including reason for exclusion)

____

____

© JBI, 2020. All rights reserved. JBI grants use of these tools for research purposes only. All other enquiries should be sent to jbisynthesis@adelaide.edu.au.

JBI CRITICAL APPRAISAL CHECKLIST FOR RANDOMIZED CONTROLLED TRIALS

Reviewer____Date________________________________

Author Tse et al., 2021 Year____________ Record Number__________________________

	Yes	No	Unclear	NA
1. Was true randomization used for assignment of participants to treatment groups?	■	☐	☐	☐
2. Was allocation to treatment groups concealed?	■	☐	☐	☐
3. Were treatment groups similar at the baseline?	■	☐	☐	☐
4. Were participants blind to treatment assignment?	☐	■	☐	☐
5. Were those delivering treatment blind to treatment assignment?	☐	■	☐	☐
6. Were outcomes assessors blind to treatment assignment?	■	☐	☐	☐
7. Were treatment groups treated identically other than the intervention of interest?	■	☐	☐	☐
8. Was follow up complete and if not, were differences between groups in terms of their follow up adequately described and analyzed?	■	☐	☐	☐
9. Were participants analyzed in the groups to which they were randomized?	■	☐	☐	☐
10. Were outcomes measured in the same way for treatment groups?	■	☐	☐	☐
11. Were outcomes measured in a reliable way?	■	☐	☐	☐
12. Was appropriate statistical analysis used?	■	☐	☐	☐
13. Was the trial design appropriate, and any deviations from the standard RCT design (individual randomization, parallel groups) accounted for in the conduct and analysis of the trial?	■	☐	☐	☐

Overall appraisal: Include ■ Exclude ☐ Seek further info ☐

Comments (Including reason for exclusion)

____

____

____

© JBI, 2020. All rights reserved. JBI grants use of these tools for research purposes only. All other enquiries should be sent to jbisynthesis@adelaide.edu.au.

JBI CRITICAL APPRAISAL CHECKLIST FOR RANDOMIZED CONTROLLED TRIALS

Reviewer______________________________________Date_______________________________

Author Tey et al., 2019 ______________________Year_____ Record Number______________________

	Yes	No	Unclear	NA
1. Was true randomization used for assignment of participants to treatment groups?	■	☐	☐	☐
2. Was allocation to treatment groups concealed?	■	☐	☐	☐
3. Were treatment groups similar at the baseline?	■	☐	☐	☐
4. Were participants blind to treatment assignment?	☐	■	☐	☐
5. Were those delivering treatment blind to treatment assignment?	☐	■	☐	☐
6. Were outcomes assessors blind to treatment assignment?	■	☐	☐	☐
7. Were treatment groups treated identically other than the intervention of interest?	■	☐	☐	☐
8. Was follow up complete and if not, were differences between groups in terms of their follow up adequately described and analyzed?	■	☐	☐	☐
9. Were participants analyzed in the groups to which they were randomized?	■	☐	☐	☐
10. Were outcomes measured in the same way for treatment groups?	■	☐	☐	☐
11. Were outcomes measured in a reliable way?	■	☐	☐	☐
12. Was appropriate statistical analysis used?	■	☐	☐	☐
13. Was the trial design appropriate, and any deviations from the standard RCT design (individual randomization, parallel groups) accounted for in the conduct and analysis of the trial?	■	☐	☐	☐

Overall appraisal: Include ■ Exclude ☐ Seek further info ☐

Comments (Including reason for exclusion)

____

____

____

© JBI, 2020. All rights reserved. JBI grants use of these tools for research purposes only. All other enquiries should be sent to jbisynthesis@adelaide.edu.au.

JBI CRITICAL APPRAISAL CHECKLIST FOR RANDOMIZED CONTROLLED TRIALS

Reviewer____Date____________________________________

Author Tan et al., 2018 Year_____________ Record Number_________________________

	Yes	No	Unclear	NA
1. Was true randomization used for assignment of participants to treatment groups?	■	☐	☐	☐
2. Was allocation to treatment groups concealed?	■	☐	☐	☐
3. Were treatment groups similar at the baseline?	■	☐	☐	☐
4. Were participants blind to treatment assignment?	☐	■	☐	☐
5. Were those delivering treatment blind to treatment assignment?	☐	■	☐	☐
6. Were outcomes assessors blind to treatment assignment?	■	☐	☐	☐
7. Were treatment groups treated identically other than the intervention of interest?	■	☐	☐	☐
8. Was follow up complete and if not, were differences between groups in terms of their follow up adequately described and analyzed?	■	☐	☐	☐
9. Were participants analyzed in the groups to which they were randomized?	■	☐	☐	☐
10. Were outcomes measured in the same way for treatment groups?	■	☐	☐	☐
11. Were outcomes measured in a reliable way?	■	☐	☐	☐
12. Was appropriate statistical analysis used?	■	☐	☐	☐
13. Was the trial design appropriate, and any deviations from the standard RCT design (individual randomization, parallel groups) accounted for in the conduct and analysis of the trial?	■	☐	☐	☐

Overall appraisal: Include ■ Exclude ☐ Seek further info ☐

Comments (Including reason for exclusion)

____

____

____

© JBI, 2020. All rights reserved. JBI grants use of these tools for research purposes only. All other enquiries should be sent to jbisynthesis@adelaide.edu.au.

JBI CRITICAL APPRAISAL CHECKLIST FOR RANDOMIZED CONTROLLED TRIALS

Reviewer____Date________________________________

Author Sun et al., 2023 Year_______________ Record Number__________________________

	Yes	No	Unclear	NA
1. Was true randomization used for assignment of participants to treatment groups?	■	☐	☐	☐
2. Was allocation to treatment groups concealed?	☐	☐	■	☐
3. Were treatment groups similar at the baseline?	■	☐	☐	☐
4. Were participants blind to treatment assignment?	☐	■	☐	☐
5. Were those delivering treatment blind to treatment assignment?	☐	☐	■	☐
6. Were outcomes assessors blind to treatment assignment?	☐	☐	■	☐
7. Were treatment groups treated identically other than the intervention of interest?	■	☐	☐	☐
8. Was follow up complete and if not, were differences between groups in terms of their follow up adequately described and analyzed?	■	☐	☐	☐
9. Were participants analyzed in the groups to which they were randomized?	■	☐	☐	☐
10. Were outcomes measured in the same way for treatment groups?	■	☐	☐	☐
11. Were outcomes measured in a reliable way?	■	☐	☐	☐
12. Was appropriate statistical analysis used?	■	☐	☐	☐
13. Was the trial design appropriate, and any deviations from the standard RCT design (individual randomization, parallel groups) accounted for in the conduct and analysis of the trial?	■	☐	☐	☐

Overall appraisal: Include ■ Exclude ☐ Seek further info ☐

Comments (Including reason for exclusion)

____

____

____

© JBI, 2020. All rights reserved. JBI grants use of these tools for research purposes only. All other enquiries should be sent to jbisynthesis@adelaide.edu.au.

JBI CRITICAL APPRAISAL CHECKLIST FOR RANDOMIZED CONTROLLED TRIALS

Reviewer____ Date__________________________________

Author Sturm., 2022 __________ Year________________ Record Number________________________

	Yes	No	Unclear	NA
1. Was true randomization used for assignment of participants to treatment groups?	■	☐	☐	☐
2. Was allocation to treatment groups concealed?	■	☐	☐	☐
3. Were treatment groups similar at the baseline?	■	☐	☐	☐
4. Were participants blind to treatment assignment?	☐	■	☐	☐
5. Were those delivering treatment blind to treatment assignment?	☐	■	☐	☐
6. Were outcomes assessors blind to treatment assignment?	☐	■	☐	☐
7. Were treatment groups treated identically other than the intervention of interest?	■	☐	☐	☐
8. Was follow up complete and if not, were differences between groups in terms of their follow up adequately described and analyzed?	■	☐	☐	☐
9. Were participants analyzed in the groups to which they were randomized?	■	☐	☐	☐
10. Were outcomes measured in the same way for treatment groups?	■	☐	☐	☐
11. Were outcomes measured in a reliable way?	■	☐	☐	☐
12. Was appropriate statistical analysis used?	■	☐	☐	☐
13. Was the trial design appropriate, and any deviations from the standard RCT design (individual randomization, parallel groups) accounted for in the conduct and analysis of the trial?	■	☐	☐	☐

Overall appraisal: Include ■ Exclude ☐ Seek further info ☐

Comments (Including reason for exclusion)

____

____

____

© JBI, 2020. All rights reserved. JBI grants use of these tools for research purposes only. All other enquiries should be sent to jbisynthesis@adelaide.edu.au.

JBI CRITICAL APPRAISAL CHECKLIST FOR RANDOMIZED CONTROLLED TRIALS

Reviewer______________________________________Date______________________________

Author Solis., 2024 Year________________ Record Number______________________

	Yes	No	Unclear	NA
1. Was true randomization used for assignment of participants to treatment groups?	■	☐	☐	☐
2. Was allocation to treatment groups concealed?	☐	☐	■	☐
3. Were treatment groups similar at the baseline?	■	☐	☐	☐
4. Were participants blind to treatment assignment?	☐	■	☐	☐
5. Were those delivering treatment blind to treatment assignment?	☐	■	☐	☐
6. Were outcomes assessors blind to treatment assignment?	■	☐	☐	☐
7. Were treatment groups treated identically other than the intervention of interest?	■	☐	☐	☐
8. Was follow up complete and if not, were differences between groups in terms of their follow up adequately described and analyzed?	■	☐	☐	☐
9. Were participants analyzed in the groups to which they were randomized?	■	☐	☐	☐
10. Were outcomes measured in the same way for treatment groups?	■	☐	☐	☐
11. Were outcomes measured in a reliable way?	■	☐	☐	☐
12. Was appropriate statistical analysis used?	■	☐	☐	☐
13. Was the trial design appropriate, and any deviations from the standard RCT design (individual randomization, parallel groups) accounted for in the conduct and analysis of the trial?	■	☐	☐	☐

Overall appraisal: Include ■ Exclude ☐ Seek further info ☐

Comments (Including reason for exclusion)

____

____

____

© JBI, 2020. All rights reserved. JBI grants use of these tools for research purposes only. All other enquiries should be sent to jbisynthesis@adelaide.edu.au.

JBI CRITICAL APPRAISAL CHECKLIST FOR RANDOMIZED CONTROLLED TRIALS

Reviewer______________________________________Date______________________________

Author Singleton., 2023 Year____________ Record Number______________________

	Yes	No	Unclear	NA
1. Was true randomization used for assignment of participants to treatment groups?	■	☐	☐	☐
2. Was allocation to treatment groups concealed?	■	☐	☐	☐
3. Were treatment groups similar at the baseline?	■	☐	☐	☐
4. Were participants blind to treatment assignment?	☐	■	☐	☐
5. Were those delivering treatment blind to treatment assignment?	☐	■	☐	☐
6. Were outcomes assessors blind to treatment assignment?	■	☐	☐	☐
7. Were treatment groups treated identically other than the intervention of interest?	■	☐	☐	☐
8. Was follow up complete and if not, were differences between groups in terms of their follow up adequately described and analyzed?	■	☐	☐	☐
9. Were participants analyzed in the groups to which they were randomized?	■	☐	☐	☐
10. Were outcomes measured in the same way for treatment groups?	■	☐	☐	☐
11. Were outcomes measured in a reliable way?	■	☐	☐	☐
12. Was appropriate statistical analysis used?	■	☐	☐	☐
13. Was the trial design appropriate, and any deviations from the standard RCT design (individual randomization, parallel groups) accounted for in the conduct and analysis of the trial?	■	☐	☐	☐

Overall appraisal: Include ■ Exclude ☐ Seek further info ☐

Comments (Including reason for exclusion)

____

____

____

© JBI, 2020. All rights reserved. JBI grants use of these tools for research purposes only. All other enquiries should be sent to jbisynthesis@adelaide.edu.au.

JBI CRITICAL APPRAISAL CHECKLIST FOR RANDOMIZED CONTROLLED TRIALS

Reviewer______________________________________Date_______________________________

Author Selzler, 2021__________Year________________ Record Number________________________

	Yes	No	Unclear	NA
1. Was true randomization used for assignment of participants to treatment groups?	■	☐	☐	☐
2. Was allocation to treatment groups concealed?	■	☐	☐	☐
3. Were treatment groups similar at the baseline?	■	☐	☐	☐
4. Were participants blind to treatment assignment?	☐	■	☐	☐
5. Were those delivering treatment blind to treatment assignment?	☐	■	☐	☐
6. Were outcomes assessors blind to treatment assignment?	■	☐	☐	☐
7. Were treatment groups treated identically other than the intervention of interest?	■	☐	☐	☐
8. Was follow up complete and if not, were differences between groups in terms of their follow up adequately described and analyzed?	■	☐	☐	☐
9. Were participants analyzed in the groups to which they were randomized?	■	☐	☐	☐
10. Were outcomes measured in the same way for treatment groups?	■	☐	☐	☐
11. Were outcomes measured in a reliable way?	■	☐	☐	☐
12. Was appropriate statistical analysis used?	■	☐	☐	☐
13. Was the trial design appropriate, and any deviations from the standard RCT design (individual randomization, parallel groups) accounted for in the conduct and analysis of the trial?	■	☐	☐	☐

Overall appraisal: Include ■ Exclude ☐ Seek further info ☐

Comments (Including reason for exclusion)

____

____

____

© JBI, 2020. All rights reserved. JBI grants use of these tools for research purposes only. All other enquiries should be sent to jbisynthesis@adelaide.edu.au.

JBI CRITICAL APPRAISAL CHECKLIST FOR RANDOMIZED CONTROLLED TRIALS

Reviewer______________________________________Date______________________________

Author Sakakibara., 2021 Year___________ Record Number______________________

	Yes	No	Unclear	NA
1. Was true randomization used for assignment of participants to treatment groups?	■	☐	☐	☐
2. Was allocation to treatment groups concealed?	■	☐	☐	☐
3. Were treatment groups similar at the baseline?	■	☐	☐	☐
4. Were participants blind to treatment assignment?	☐	■	☐	☐
5. Were those delivering treatment blind to treatment assignment?	☐	■	☐	☐
6. Were outcomes assessors blind to treatment assignment?	■	☐	☐	☐
7. Were treatment groups treated identically other than the intervention of interest?	■	☐	☐	☐
8. Was follow up complete and if not, were differences between groups in terms of their follow up adequately described and analyzed?	■	☐	☐	☐
9. Were participants analyzed in the groups to which they were randomized?	■	☐	☐	☐
10. Were outcomes measured in the same way for treatment groups?	■	☐	☐	☐
11. Were outcomes measured in a reliable way?	■	☐	☐	☐
12. Was appropriate statistical analysis used?	■	☐	☐	☐
13. Was the trial design appropriate, and any deviations from the standard RCT design (individual randomization, parallel groups) accounted for in the conduct and analysis of the trial?	■	☐	☐	☐

Overall appraisal: Include ■ Exclude ☐ Seek further info ☐

Comments (Including reason for exclusion)

© JBI, 2020. All rights reserved. JBI grants use of these tools for research purposes only. All other enquiries should be sent to jbisynthesis@adelaide.edu.au.

JBI CRITICAL APPRAISAL CHECKLIST FOR RANDOMIZED CONTROLLED TRIALS

Reviewer____Date__________________________________

Author Saghaee et al., 2020__________Year__________ Record Number_________________________

	Yes	No	Unclear	NA
1. Was true randomization used for assignment of participants to treatment groups?	■	☐	☐	☐
2. Was allocation to treatment groups concealed?	■	☐	☐	☐
3. Were treatment groups similar at the baseline?	■	☐	☐	☐
4. Were participants blind to treatment assignment?	☐	■	☐	☐
5. Were those delivering treatment blind to treatment assignment?	☐	■	☐	☐
6. Were outcomes assessors blind to treatment assignment?	■	☐	☐	☐
7. Were treatment groups treated identically other than the intervention of interest?	■	☐	☐	☐
8. Was follow up complete and if not, were differences between groups in terms of their follow up adequately described and analyzed?	■	☐	☐	☐
9. Were participants analyzed in the groups to which they were randomized?	■	☐	☐	☐
10. Were outcomes measured in the same way for treatment groups?	■	☐	☐	☐
11. Were outcomes measured in a reliable way?	■	☐	☐	☐
12. Was appropriate statistical analysis used?	■	☐	☐	☐
13. Was the trial design appropriate, and any deviations from the standard RCT design (individual randomization, parallel groups) accounted for in the conduct and analysis of the trial?	■	☐	☐	☐

Overall appraisal: Include ■ Exclude ☐ Seek further info ☐

Comments (Including reason for exclusion)

____

____

____

© JBI, 2020. All rights reserved. JBI grants use of these tools for research purposes only. All other enquiries should be sent to jbisynthesis@adelaide.edu.au.

JBI CRITICAL APPRAISAL CHECKLIST FOR RANDOMIZED CONTROLLED TRIALS

Reviewer______________________________________Date_______________________________

Author Rahimi., 2020 __________ Year______________ Record Number______________________

	Yes	No	Unclear	NA
1. Was true randomization used for assignment of participants to treatment groups?	■	☐	☐	☐
2. Was allocation to treatment groups concealed?	■	☐	☐	☐
3. Were treatment groups similar at the baseline?	■	☐	☐	☐
4. Were participants blind to treatment assignment?	■	☐	☐	☐
5. Were those delivering treatment blind to treatment assignment?	☐	■	☐	☐
6. Were outcomes assessors blind to treatment assignment?	■	☐	☐	☐
7. Were treatment groups treated identically other than the intervention of interest?	■	☐	☐	☐
8. Was follow up complete and if not, were differences between groups in terms of their follow up adequately described and analyzed?	■	☐	☐	☐
9. Were participants analyzed in the groups to which they were randomized?	■	☐	☐	☐
10. Were outcomes measured in the same way for treatment groups?	■	☐	☐	☐
11. Were outcomes measured in a reliable way?	■	☐	☐	☐
12. Was appropriate statistical analysis used?	■	☐	☐	☐
13. Was the trial design appropriate, and any deviations from the standard RCT design (individual randomization, parallel groups) accounted for in the conduct and analysis of the trial?	■	☐	☐	☐

Overall appraisal: Include ■ Exclude ☐ Seek further info ☐

Comments (Including reason for exclusion)

____

____

© JBI, 2020. All rights reserved. JBI grants use of these tools for research purposes only. All other enquiries should be sent to jbisynthesis@adelaide.edu.au.

JBI CRITICAL APPRAISAL CHECKLIST FOR RANDOMIZED CONTROLLED TRIALS

Reviewer____Date________________________________

Author Portz., 2019 Year_________________ Record Number_________________________

	Yes	No	Unclear	NA
1. Was true randomization used for assignment of participants to treatment groups?	■	☐	☐	☐
2. Was allocation to treatment groups concealed?	☐	☐	■	☐
3. Were treatment groups similar at the baseline?	■	☐	☐	☐
4. Were participants blind to treatment assignment?	☐	■	☐	☐
5. Were those delivering treatment blind to treatment assignment?	☐	■	☐	☐
6. Were outcomes assessors blind to treatment assignment?	☐	☐	■	☐
7. Were treatment groups treated identically other than the intervention of interest?	■	☐	☐	☐
8. Was follow up complete and if not, were differences between groups in terms of their follow up adequately described and analyzed?	■	☐	☐	☐
9. Were participants analyzed in the groups to which they were randomized?	■	☐	☐	☐
10. Were outcomes measured in the same way for treatment groups?	■	☐	☐	☐
11. Were outcomes measured in a reliable way?	■	☐	☐	☐
12. Was appropriate statistical analysis used?	■	☐	☐	☐
13. Was the trial design appropriate, and any deviations from the standard RCT design (individual randomization, parallel groups) accounted for in the conduct and analysis of the trial?	■	☐	☐	☐

Overall appraisal: Include ■ Exclude ☐ Seek further info ☐

Comments (Including reason for exclusion)

____

____

____

© JBI, 2020. All rights reserved. JBI grants use of these tools for research purposes only. All other enquiries should be sent to jbisynthesis@adelaide.edu.au.
